# Apolipoprotein E mimetic peptide, CN‐105, improves outcomes in ischemic stroke

**DOI:** 10.1002/acn3.399

**Published:** 2017-03-09

**Authors:** Tian Ming Tu, Brad J. Kolls, Erik J. Soderblom, Viviana Cantillana, Paul Durham Ferrell, M. Arthur Moseley, Haichen Wang, Hana N. Dawson, Daniel T. Laskowitz

**Affiliations:** ^1^ Department of NeurologyDuke University School of MedicineDurhamNorth Carolina; ^2^ Department of NeurologyNational Neuroscience InstituteTan Tock Seng CampusSingapore; ^3^ Duke Proteomics Core FacilityCenter for Genomic and Computational BiologyDuke UniversityDurhamNorth Carolina; ^4^ Department of PathologyDuke University School of MedicineDurhamNorth Carolina

## Abstract

**Objective:**

At present, the absence of a pharmacological neuroprotectant represents an important unmet clinical need in the treatment of ischemic and traumatic brain injury. Recent evidence suggests that administration of apolipoprotein E mimetic therapies represent a viable therapeutic strategy in this setting. We investigate the neuroprotective and anti‐inflammatory properties of the apolipoprotein E mimetic pentapeptide, CN‐105, in a microglial cell line and murine model of ischemic stroke.

**Methods:**

Ten to 13‐week‐old male C57/BL6 mice underwent transient middle cerebral artery occlusion and were randomly selected to receive CN‐105 (0.1 mg/kg) in 100 *μ*L volume or vehicle via tail vein injection at various time points. Survival, motor‐sensory functional outcomes using rotarod test and 4‐limb wire hanging test, infarct volume assessment using 2,3,5‐Triphenyltetrazolium chloride staining method, and microglial activation in the contralateral hippocampus using F4/80 immunostaining were assessed at various time points. In vitro assessment of tumor necrosis factor‐alpha secretion in a microglial cell line was performed, and phosphoproteomic analysis conducted to explore early mechanistic pathways of CN‐105 in ischemic stroke.

**Results:**

Mice receiving CN‐105 demonstrated improved survival, improved functional outcomes, reduced infarct volume, and reduced microglial activation. CN‐105 also suppressed inflammatory cytokines secretion in microglial cells in vitro. Phosphoproteomic signals suggest that CN‐105 reduces proinflammatory pathways and lower oxidative stress.

**Interpretation:**

CN‐105 improves functional and histological outcomes in a murine model of ischemic stroke via modulation of neuroinflammatory pathways. Recent clinical trial of this compound has demonstrated favorable pharmacokinetic and safety profile, suggesting that CN‐105 represents an attractive candidate for clinical translation.

## Introduction

Ischemic stroke is a major cause of morbidity and mortality worldwide.^1^, ^2^ Currently, the only approved treatments for acute ischemic stroke include thrombolysis and endovascular thrombectomy.^3^ Because of the stringent time criteria to qualify for these treatments,^4^ <7% of all ischemic stroke patients actually receive these reperfusion interventions.^5^, ^6^, ^7^ Unfortunately, no other neuroprotective agent has been demonstrated to improve outcome. Thus, the development of a pharmacological intervention that mitigates secondary neuronal injury remains a compelling unmet clinical need.

The postischemic brain demonstrates a proinflammatory cascade, characterized by glial activation and release inflammatory mediators, neuronal excitotoxicity, and oxidative stress.^8^, ^9^ This neuroinflammatory cascade begins immediately after vascular occlusion and exacerbates secondary neuronal injury, resulting in further tissue injury days after the initial insult.^9^, ^10^, ^11^, ^12^ Targeting this cascade has potential for reducing tissue injury and lengthening the therapeutic window for revascularization. However, drugs targeting the inflammatory cascade have been unsuccessful in clinical trials, notably due to adverse effects and poor central nervous system (CNS) penetration.^13^, ^14^

Apolipoprotein E (apoE) is the primary apolipoprotein produced in the CNS, where its glial secretion is upregulated after injury.^15^ ApoE has demonstrated to suppress microglial and astrocyte activation in in vitro^16^, ^17^, ^18^ and in vivo models of brain injury.^19^, ^20^ In addition to its immunomodulatory effects, apoE attenuates N‐methyl‐D‐aspartate‐mediated neuronal excitotoxicity in cell culture models.^21^ ApoE also appears to protect from cerebral ischemia as apoE‐deficient animals demonstrate worse outcomes, both functionally and histologically, following experimental ischemia.^19^, ^22^, ^23^ ApoE exists in humans as three common isoforms, differing by single amino acid substitutions at residues 112 and 158.^24^ Isoform‐specific protective effects on endogenous neuroinflammatory responses have been observed in humans across different acute brain injuries^25^, ^26^ but not in cerebral ischemia.^27^, ^28^ Due to its large size, apoE does not cross the blood–brain barrier (BBB) and its therapeutic potential is limited. To overcome this limitation, smaller apoE‐mimetic peptides, derived from the helical receptor binding region of apoE, have been developed that retain the functional effects of the holoprotein on receptor binding^29^ in reducing inflammation^17^ and neuronal excitotoxicity.^30^ Moreover, these apoE‐mimetic compounds have demonstrated long‐term functional and histological improvements in preclinical models of ischemic stroke^31^, ^32^ and numerous other acute CNS injuries^33^, ^34^, ^35^, ^36^, ^37^, ^38^, ^39^, ^40^, ^41^, ^42^, ^43^, ^44^, ^45^, ^46^ (Table 1).

**Table 1:** Therapeutic efficacy of apoE‐mimetic peptides in preclinical models of acute brain injuries

Injury	Species	ApoE‐mimetic peptide	Functional improvement and survival advantage	Histological improvement	Biochemical improvement	References
Ischemic Stroke (tMCAO)	Sprague–Dawley rats	COG1410	Improved vestibulomotor (7 days), locomotor function	Reduction in infarct volume (35 days)	–	Tukhouvskaya et al., 2009
	C57Bl/6J mice	COG1410	Improved vestibulomotor function (3 days)	Reduction in infarct volume (24 h), cerebral edema	Reduced of inflammatory cytokine (TNF‐*α* RNA)	Wang et al., 2013
Perinatal Hypoxia‐Ischemia	Wistar rat pups	COG133	Reduced mortality	Reduction in brain tissue loss	–	McAdoo et al., 2005
Intracerebral hemorrhage (collagenase injection)	C57Bl/6J mice	COG1410	Improved vestibulomotor function (7 days)	Reduction in cerebral edema	Reduction in inflammatory cytokines (IL‐6 and eNOS)	James et al., 2009
	C57/BL6 mice	COG1410	Improved vestibulomotor function (5 days), survival and reduced neurological severity.	Reduction in microgliosis, cerebral edema, and neuronal injury	Reduction in inflammatory signaling (phosphorylated p38 and NF‐kB protein)	Laskowitz et al., 2012
	C57/BL6 mice	CN‐105	Improved vestibulomotor function (5 days) and memory	Reduction in cerebral edema, microgliosis, and neuronal degeneration	–	Lei et al., 2016
Subarachnoid hemorrhage	C57/BL6 mice	COG1410	Improved vestibulomotor function and reduced neurological severity	Reduction in microgliosis	Reduction in apoptotic markers and inflammatory signals (JNK, c‐Jun and p65)	Wu et al., 2016
	C57/BL6 mice	COG1410	Improved vestibulomotor function (3 days), survival and reduced neurological severity	Reduction in vasospasm and cerebral edema	–	Gao et al., 2006
	C57/BL6 mice	COG1410	Improved vestibulomotor function (3 days)	Reduction in vasospasm		Mesis et al., 2006
Traumatic brain injury (closed head injury)	C57Bl/6J mice	COG133	Improved vestibulomotor function and memory (5 days)	Reduction in neuronal degeneration	Reduction in oxidative stress (aconitase) and inflammatory cytokine (TNF*α*)	Lynch et al., 2005
	C57Bl/6J mice	COG133	Improved vestibulomotor function (5 days)	Reduction in neuronal degeneration and microgliosis	Reduction in amyloid‐beta expression	Wang et al., 2007
	C57Bl/6J mice	COG1410	Improved vestibulomotor function (5 days) and memory	Reduction in neuronal degeneration and microgliosis	–	Laskowitz et al., 2007
Traumatic brain injury (controlled cortical impact)	Sprague–Dawley rats	COG1410	Improved sensorimotor function (14 days)	Reduction in lesion volume and astrocytosis	–	Hoane et al., 2007
	Sprague–Dawley rats	COG1410	Improved somatosensory function and memory	Reduction in neuronal degeneration	–	Hoane et al., 2009
	C57Bl/6J mice	COG1410	Improved vestibulomotor (3 days) and neurological function	Reduction in lesion volume, cerebral edema, and BBB disruption	–	Cao et al., 2016
	C57Bl/6J mice	COG1410	Improved vestibulomotor function (7 days)	Reduction in cerebral edema, microvascular density, and neuronal degeneration	Reduction in VEGF expression and increased brain glucose uptake	Qin et al., 2016

BBB, blood–brain barrier; eNOS, endothelial nitric oxide synthase; IL‐6, interleukin‐6; JNK, c‐Jun N‐terminal kinases; NF‐kB, nuclear factor kappa‐light‐chain‐enhancer of activated B cells; tMCAO, transient middle cerebral artery occlusion; TNF‐*α*, tumor necrosis factor alpha; VEGF, vascular endothelial growth factor.

CN‐105 is an apoE‐mimetic 5 amino acid peptide (acetyl‐Valine‐Serine‐Arginine‐Arginine‐Arginine‐amide) derived from the apoE receptor binding region by linearizing the polar face of the amphipathic helix involved in receptor interaction.^35^ CN‐105 has the advantage of increased CNS penetration^35^ over previous larger apoE‐mimetic peptides and has demonstrated efficacy in experimental intracerebral hemorrhage.^35^ Based on its preclinical safety and efficacy, CN‐105 was granted a successful investigational new drug application by the United States Food and Drug Administration and recently completed a Phase 1 clinical trial (clinical trials identifier: NCT02670824), where safety was demonstrated in both single escalating dose and multiple dosing paradigms.^47^ The evidence of good CNS penetration, efficacy in acute brain injury models, and reassuring safety profile suggest CN‐105 may be a viable candidate drug for further clinical development as a neuroprotective agent.

This study evaluates the therapeutic efficacy of CN‐105 in a murine model of transient focal cerebral ischemia and reperfusion. We test the hypothesis that intravenous administration of CN‐105 reduces mortality, results in improvement of functional and histological outcomes after experimental ischemic stroke, and reduces microglial activation. We also explore the mechanisms by which CN‐105 exerts its immunomodulatory and neuroprotective effects by performing a phosphoproteomic analysis in animals exposed to cerebral ischemia.

## Materials and Methods

### Physiological effects of CN‐105

We performed a series of experiments to assess the safety and determine the physiological effects of CN‐105 using both males and females 8‐week‐old Crl:CD(SD) rats (Charles River Laboratories, Portage, Michigan). These experiments were independently conducted (MPI Research Inc, Mattawan, Michigan) prior to the conduct of further experiments in this study. These animals received an implanted femoral vein catheter exteriorized between the scapulae for intravenous dosing purposes. 0.9% Sodium Chloride for injection was used as vehicle. CN‐105 was synthesized by Polypeptide Inc (San Diego, CA) to a purity of >99%. CN‐105 was dissolved in vehicle to the concentration of 1.25 mg/mL. Repeated intravenous administration of 5 mg CN‐105/kg/dose or vehicle, four times per day (20 mg/kg/day) for 14 days 6 h apart (total of 56 doses per animal). Compared to the subsequent experiments, doses of CN‐105 used in these physiological studies were 50 times higher per dose and 200 times higher per day. Animals were randomized prior to any experiments and outcome assessments were performed in a blinded fashion.

Multiple physiological parameters were measured in these experiments. These included body temperature, hematological (platelet count and coagulation profile), biochemistry (glucose, creatinine, and total bilirubin), and respiratory (respiratory rate and minute ventilation) evaluations. Full experimental details are available in Data S1.

### Peptide synthesis and administration

CN‐105 used in subsequent animal experiments was similarly synthesized by Polypeptide Inc (San Diego, CA) to a purity of >99%. However, CN‐105 was dissolved in 1× phosphate‐buffered saline (PBS), reconstituted to 0.1 mg/kg concentration in 100 *μ*L volume and administered via tail vein injection.

### Animal procedures

In all experiments, mice were randomized to treatment and vehicle group before injury and all animals were treated with allocation concealment. In each experiment, mice were randomized to treatment or vehicle groups prior to injury. Animals were treated with blinded concealment. All procedures and assessments were performed in blinded fashion. Animal procedures were approved by the Duke University Institutional Animal Care and Use Committee in keeping with established guidelines.

### Model of focal ischemia and reperfusion

Ten‐ to 13‐week‐old C57Bl/6J male mice (Jackson Laboratories, Bar Harbor, ME) were used. Focal cerebral ischemia was induced by filamentous transient middle cerebral artery occlusion (tMCAO), as previously described.^32^ To model the effect of CN‐105 in different clinical stroke severities, two different ischemic intervals were used. A 30‐min ischemic occlusion associated with high mortality was used to assess for mortality advantage and the phosphoproteomic signature of CN‐105 effects on large stroke volumes. A shorter 15‐min ischemic occlusion time, associated with lower mortality, was used to assess long‐term functional and histological endpoints.

#### Functional assessments

Vestibulomotor performance was assessed daily postinjury with the accelerating rotarod (Med Associates Inc, Georgia, Vermont), as previously described.^32^ The time in which the mouse was able to stay on the rotating rod before falling (rotarod latency) was recorded up to 300 sec and the average of three trials was used.

Motor coordination and limb muscle strength was assessed with the 4‐limb wire hanging test.^48^ A mouse was placed on the center of a wire grid upright and the grid was inverted gently. The time it took for the mouse to fall off the grid (hanging latency) was recorded up to 600 sec. The test was performed at 2 and 7 days post injury and repeated three times per day. Maximal hanging latency was used.

#### Infarct volume measurements

Coronally sectioned (1‐mm thick) brain slices were stained in 2% 2,3,5‐triphenyletrazolium chloride (Sigma, St. Louis, MO) according to protocol.^49^ After staining, images were captured and analyzed using open‐source software (Image J, version 1.49, National Institutes of Health, Bethesda, Maryland). The infarcted area per slice was determined by subtracting the area of viable stained tissue of the ipsilateral hemisphere from the unaffected viable area of the contralateral hemisphere. Final infarct volume was the sum of infarcted areas multiplied by slice thickness.

#### Contralateral hemisphere microglial quantification

Frozen coronal brain sections (40 *μ*m thick) were stained with anti‐rat F4/80 antibody (rat monoclonal, 1:10,000; Serotec, Raleigh, NC) specific for activated microglia and cells of the monocyte lineage.^34^ Microglial count was performed in the contralateral hippocampus using the optical fractionator method.^50^ Microglia per unit volume (count density) was used to allow comparability between animals.

### TNF‐*α* suppression by CN‐105 in microglial cells

For all in vitro experiments, C8‐B4 mouse microglia cells (ATCC, Manassas, VA) were used. We investigated the change in production of tumor necrosis factor‐alpha (TNF‐*α*) produced by increasing concentrations of CN‐105 (0.1, 0.3, and 1 *μ*M/mL) using fixed lipopolysaccharide (LPS) (Sigma‐Aldrich, St. Louis, MO) stimulation (10 ng/mL), and by fixed concentration of 1 *μ*M/mL of CN‐105 using increasing concentrations of LPS stimulation (10, 50, and 100 ng/mL). Cell supernatant was analyzed using Mouse TNF‐*α* DuoSet ELISA kit (R&D Systems, Minneapolis, MN) per manufacturer's protocol. All experiments were repeated six times and mean TNF‐*α* concentrations were recorded at 4 h post incubation.

### Differential phosphopeptide expression

Following 30 min of ischemia, CN‐105 or vehicle was administered at 15 min after reperfusion. At 30 min after reperfusion, mice were killed, brains were removed and dissected in the midsagittal plane. Each hemisphere was flash‐frozen separately in liquid nitrogen and stored at −80°C. The injured right hemispheres were used for analysis. Peptides were prepared from brain samples for liquid chromatography–tandem mass spectrometry (LC‐MS/MS) analysis (Data S2). Peptide digests obtained from each of the samples were analyzed in a label‐free quantitative fashion using a nanoAcquity UPLC system coupled to a Synapt HDMS mass spectrometer (Waters Corp, Milford, MA) for unenriched peptide analyses and an LTQ Orbitrap XL (Thermo Fisher Scientific, Waltham, MA) for phosphopeptide analyses. Robust peak detection and label‐free alignment of individual peptides across all sample injections was performed using the commercial package Rosetta Elucidator v3.3 (Rosetta Biosoftware, Inc., Seattle, WA) with PeakTeller algorithm.

### Statistical analysis

Independent two‐tailed *t*‐test was performed for infarct volume, microglial density analysis, and TNF‐*α* concentration of microglial cells. Repeated measures analysis of variance (ANOVA) was performed for rotarod and 4‐limb wire hanging latencies, and Dunnett's post hoc test for multiple comparisons was used. Log‐rank test was performed for survival evaluation. Principal components analysis was performed between sample groups to screen for outliers in the phosphoproteomic data. The independent two‐tailed *t*‐test was performed on log^2^ protein intensities of the phosphopeptide (peptide‐level) datasets. Significance level for all tests was set at *P* < 0.05. All data were analyzed using SPSS version 20 (IBM, Armonk, NY).

## Results

### Absence of adverse physiological effects by CN‐105

Repeated intravenous administration of 5 mg CN‐105/kg/dose 4× per day (20 mg/kg/day) for 14 days over 5 min did not produce mortality and was without significant clinical effect on body temperature, hematology and coagulation, biochemistry, and respiratory parameters. Systemic exposure to CN‐105 was independent of sex and did not appear to change following repeated administration. Full results on physiological testing of CN‐105 are found in Supplemental Material 1.

### CN‐105 confers survival benefit and functional improvements

**Figure 1:**
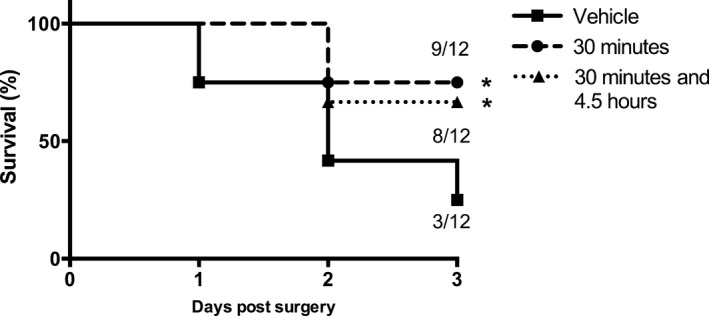
CN‐105 confers survival benefit in ischemic stroke. Intravenous CN‐105 confers survival benefit at 3 days (A) when administered via a single dose at 30 min post reperfusion, and via 2 doses at 30 min and 4.5 h post reperfusion, utilizing the murine model of transient middle cerebral artery occlusion (tMCAO) of 30 min ischemic occlusion time. (*P < 0.05 by log‐rank test compared to vehicle).

**Figure 2:**
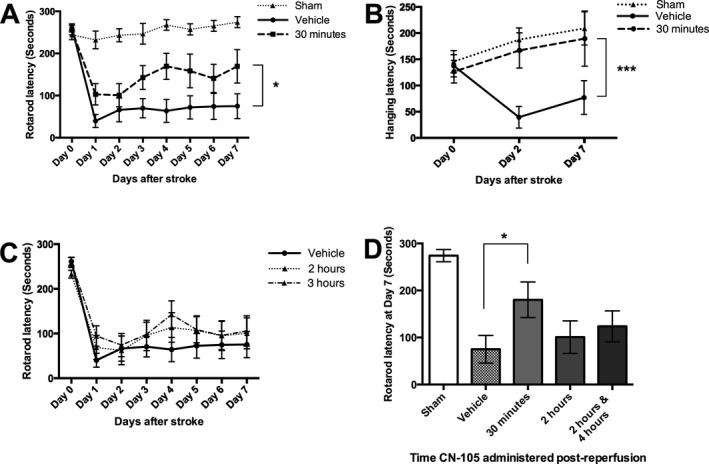
CN‐105 improves functional outcomes in ischemic stroke. (A) Improvement in rotarod latency when CN‐105 is administered 30 min post reperfusion, compared to vehicle. Improvement is observed from first day post stroke and increase over the subsequent 7 days. (B) Improvement in the 4‐limb wire hanging test latency when CN‐105 is administered 30 min post reperfusion, compared to vehicle. Improvement is observed from day 2 post‐stroke and maintained till day 7. (C) No improvement of rotarod timing observed when CN‐105 is administered 2 and 3 h post reperfusion. (D) Rotarod latency at day 7 for all treatment groups. (*P < 0.05 and ***P < 0.001 by repeated measures ANOVA and *P < 0.05 by independent *t*‐test, and error bars represent standard error of mean).

CN‐105 administrated at 30‐min post reperfusion, compared to vehicle, significantly improved survival at 3 days (75% vs. 25%, *P* = 0.013) (Fig. 1). An additional dose of CN‐105 at 4.5 h after reperfusion maintained the survival benefit, but did not enhance it. Administration of CN‐105 also conferred functional improvement following ischemic injury as demonstrated by improved rotarod latency (*P* = 0.035) and hanging latency (*P* < 0.001). The improvement was observed as early as the first day post injury and the benefit was maintained up to 7 days. No significant functional improvement was observed when CN‐105 was administered beyond 30 min post reperfusion (Fig. 2).

### CN‐105 reduces infarct volumes and contralateral microglial activation

**Figure 3:**
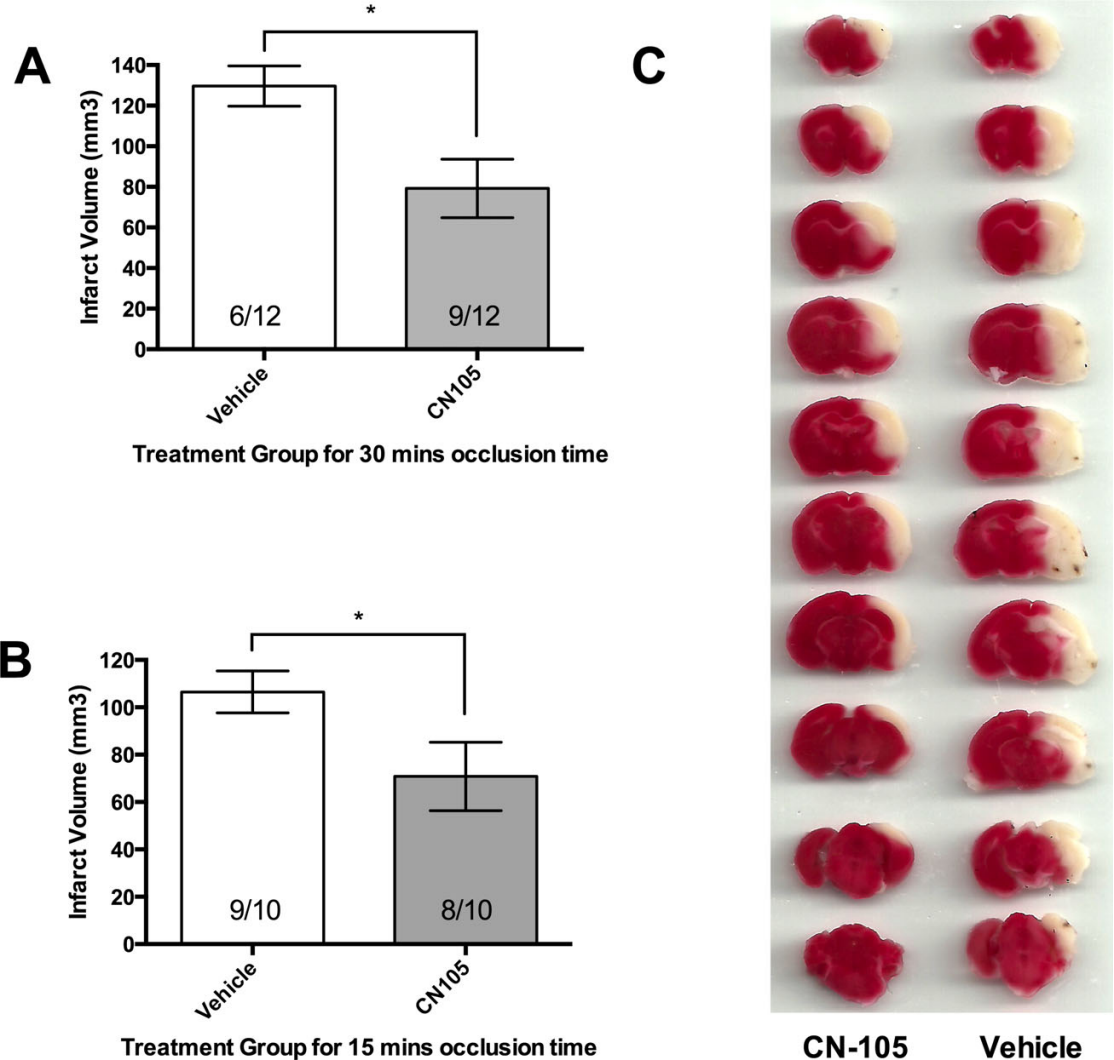
Significant reduction in infarct volume at 72 h post stroke using 2% 2,3,5‐triphenyletrazolium chloride staining methods. Reduction was observed in both our murine transient middle cerebral artery occlusion models of (A) 30 min ischemic occlusion time and (B) 15 min ischemic occlusion time. Numbers indicate the number of mice that survived to 72 h in each experimental group. (C) Representative brain slices from the 30 min ischemic occlusion experiment demonstrating the reduction in infarct volume with CN‐105. (*P < 0.05 by two‐tailed independent *t*‐test and error bars represent standard error of mean).

CN‐105, when administered 30 min post reperfusion, demonstrated a significant reduction in infarct volume at 72 h post stroke. The reduction in infarct volume was observed in both our tMCAO models of 30 min (126.7 ± 9.9 mm^3^ vs. 79.2 ± 14.4 mm^3^, *P* = 0.023) and 15 min ischemic occlusion time (106.5 ± 8.8 mm^3^ vs. 70.8 ± 14.5 mm^3^, *P* = 0.048) (Fig. 3). This represents an approximate 38% and 34% reduction in infarct volume in the 30 min and 15 min models, respectively.

We observed that the survival in the vehicle arm in this experiment with 30‐min ischemic occlusion‐time (6 of 12 mice) was better than the preceding mortality experiment (3 of 12 mice). However, the survival of the CN‐105 arm was the same for both experiments (9 of 12 mice). Moreover, a post hoc analysis of survival at 72‐h, by combining the survival data of both experiments and using a two‐tailed chi‐squared test, still demonstrated significant survival benefit (15/24 (62.5%) vs. 9/24 (37.5%), *P* = 0.0189), supporting the mortality benefit of CN‐105.

**Figure 4:**
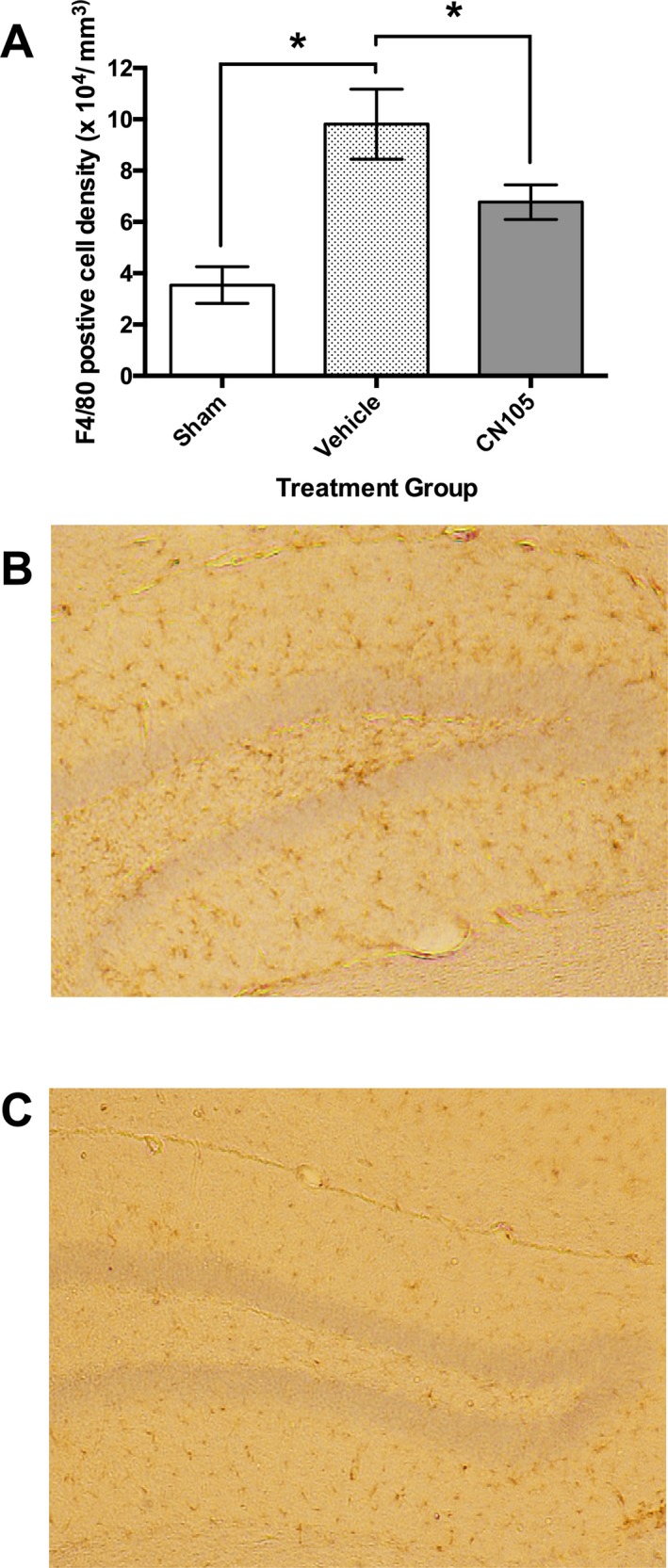
F4/80 immunocytochemical staining of microglia in contralateral hippocampus 7 days after ischemic stroke. (A) Microglial count density was significantly reduced by CN‐105, when administered 30 min post reperfusion, as compared to vehicle. Numbers indicate the number of mice that survived to 7 days in each experimental group. Representative images (4× magnification) of contralateral hippocampus demonstrating reduction in microglial cell density in hippocampal sections treated with vehicle (B) and CN‐105 (C). Microglial cells are stained brown by anti‐rat F4/80 antibody within the hippocampus. (*P < 0.05 by two‐tailed independent *t*‐test and error bars represent standard error of mean).

To assess the global protective effects from neuroinflammation by CN‐105 in focal ischemia, we quantified the amount of microglial activation in the contralateral uninjured hippocampus at 7 days post injury, following a 15‐min ischemic interval. Stereological analysis revealed significant increase in F4/80 immunopositive microglial cell density in tMCAO vehicle‐treated mice versus sham (9.8 ± 1.4 × 10^4^/mm^3^ vs. 3.5 ± 0.7 × 10^4^/mm^3^, *P* = 0.016). There was significant reduction in microglial cell density by CN‐105 compared to vehicle (6.8 ± 0.7 × 10^4^/mm^3^ vs. 9.8 ± 1.4 × 10^4^/mm^3^, *P* = 0.047) (Fig. 4).

### CN‐105 suppresses TNF‐*α* production in LPS‐stimulated microglial cells

**Figure 5:**
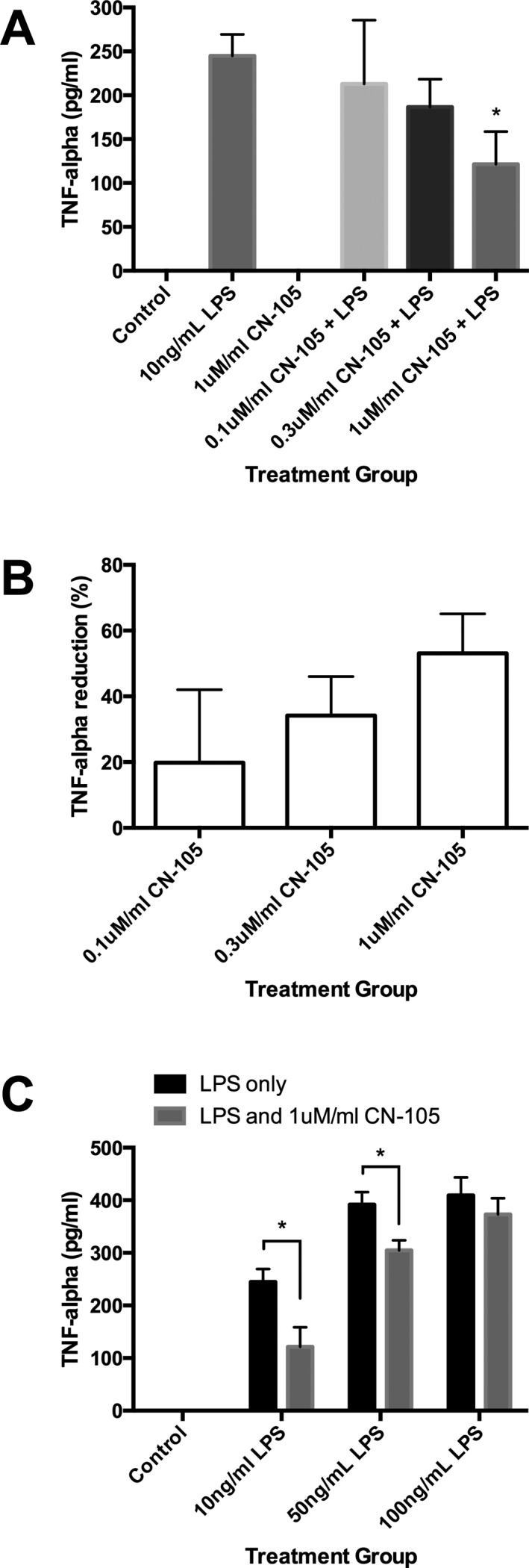
CN‐105 induced suppression of microglial tumor necrosis factor alpha (TNF‐*α*) secretion. C8‐B4 microglial cells were incubated with LPS and subsequent TNF‐*α* production was quantified by enzyme‐linked immunosorbent assay (ELISA). (A) TNF‐*α* was measured at 4 h after incubation. (B) Percentage suppression of TNF‐*α* secretion using 10 ng/mL LPS measured at 4 h post incubation with 0.1, 0.3, and 1 *μ*M/mL of CN‐105 compared to LPS alone. (C) TNF‐*α* secretion at 10, 50, 100 ng/mL LPS with 1 *μ*mol/L of CN‐105. (*P < 0.05 by two‐tailed independent *t*‐test and error bars represent standard error of mean) LPS, lipopolysaccharide.

In cultures of C8‐B4 murine microglial cells, TNF‐*α* production was increased in response to LPS stimulation. There was significant suppression of microglial TNF‐*α* production in the presence of 1 *μ*M/mL concentration of CN‐105 versus LPS alone (121.5 ± 37 ng/mL vs. 245 ± 25 ng/mL, *P* = 0.0196) using 10 ng/ml LPS stimulation measured at 4 h. Although suppression of TNF‐*α* production was not significant at lower concentrations of CN‐105, the percentage of suppression of TNF‐*α* by CN‐105 is concentration‐dependent with 20%, 34% and 53% suppression observed with 0.1, 0.3 and 1 *μ*M/mL of CN‐105, respectively. CN‐105 was also able to suppress microglial TNF‐*α* secretion up to a concentration of 50 ng/mL of LPS stimulation (Fig. 5).

### Phosphoproteomic signatures support neuroprotective effects of CN‐105

To explore potential pathways by which CN‐105 exerted its neuroprotective effects, we performed a phosphoproteomic analysis of the ischemic brain. Protein phosphorylation is critical to cell signaling, in which the binding of ligand to an extracellular domain may modify multiple downstream cell functions.^51^

**Figure 6:**
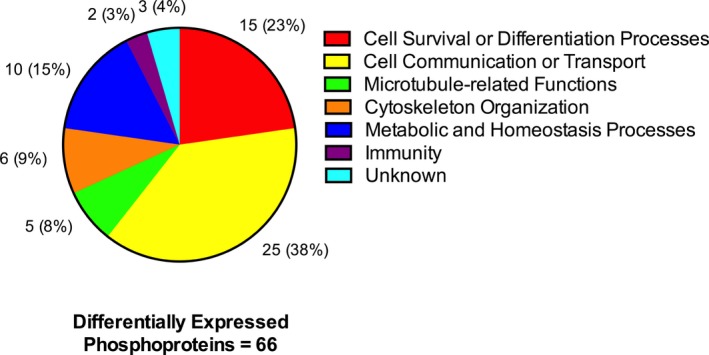
Main biological processes of differentially expressed phosphoproteins by CN‐105 in ischemic stroke. Sixty‐six phosphoproteins were differentially expressed between CN‐105 and vehicle in ischemic stroke. Majority of the biological functions of these phosphoproteins were involved in cell survival and signaling.

CN‐105 affected phosphorylation of neuroinflammatory proteins in ischemic stroke. Seventy‐one unique phosphotyrosine sites were differentially changed between CN‐105 and vehicle, of which 6 were upregulated and 65 were downregulated by more than twofold. These 71 phosphotyrosine sites corresponded to 66 unique phosphoproteins. Notable phosphoproteins differentially changed by CN‐105 mediate cellular survival (Rho GTPase activating protein 1), immunity (tyrosine kinase Lyn), and BBB integrity (cyclophilin A) (Table 2). Biological processes altered by CN‐105 (Fig. 6 and Table S1), known signaling pathways, biological functions of each phosphoproteins, and results of enrichment specificity, data quality control, and outlier screening can be found in the supplementary material (Table S2–S3 and Data S2).

**Table 2:** Statistically different phosphopeptides between CN‐105 and vehicle in ischemic stroke

Protein Name	Gene symbol and UniProtKB number	Modified peptide sequence^a^ (phosphotyrosine position)	CN‐105 v vehicle fold change	*P* ‐value (two‐tailed *t*‐test)	Biological process of protein	Function of specific phosphorylation site
**Upregulated Phosphopeptides**						
Disks large‐associated protein 2	Dlgap2, Q8BJ42	MH**Y**SSHYDTR (169)	2.416	0.040	May play a role in the molecular organization of synapses and neuronal cell signaling.	Unknown
Golgi integral membrane protein 4	Golim4, Q8BXA1	GRQEH**Y**EEEEDEEDGAAVAEK (633)	2.039	0.037	Plays a role in endosome to Golgi protein trafficking	Controlled by leptin in mouse liver.
Histone H4	Hist1h4a, P62806	KTVTAMDVV**Y**ALK (88)	2.473	0.036	Chromatin organization	Unknown
		TVTAMDVV**Y**ALK (88)	2.057	0.026		
Insulin receptor substrate 2	Irs2, P81122	SDD**Y**MPMSPTSVSAPK (671)	13.414	0.048	Transmembrane receptor protein tyrosine kinase signaling	Insulin induce phosphorylation at Y671, which in turn activate PI3K/Akt/eNOS pathway in endothelial cells.
Probable phospholipid‐transporting ATPase IA	Atp8a1, P70704	NTQWVHGIVV**Y**TGHDTK (269)	9.686	0.020	Anion transport, Catabolic process	Unknown
Sodium/potassium‐transporting ATPase subunit alpha‐1	Atp1a1, Q8VDN2	K**Y**GTDLSR (55)	2.259	0.036	Homeostatic process, Catabolic process	Unknown
**Downregulated Phosphopeptides**						
1‐phosphatidylinositol‐4,5‐bisphosphate phosphodiesterase gamma‐1	Plcg1, Q62077	KLAEGSA**Y**EEVPTSVMYSENDISNSIK (472)	−3.211	0.043	Intracellular signal transduction via G‐protein‐coupled receptor signaling and Phospholipid metabolic process	Human analog controlled by ephrin_B1
14‐3‐3 protein beta/alpha	Ywhab, Q9CQV8	QTTVSNSQQA**Y**QEAFEISKK (151)	−2.391	0.037	Cell cycle	Unknown
		QTTVSNSQQA**Y**QEAFEISK (151)	−2.434	0.028		
Alpha‐enolase	Eno1, P17182	SGK**Y**DLDFK (257)	−2.078	0.035	Glycolysis	Unknown
Ankyrin‐2	Ank2, Q8C8R3	NG**Y**TPLHIAAK (630)	−2.338	0.010	Protein localization and Intracellular protein transport	Unknown
		QELEDNDK**Y**QQFR (2113)	−6.332	0.014		Novel phosphorylation site
Band 4.1‐like protein 1	Epb41l1, Q9Z2H5	HLTQQDTRPAEQSLDMDDKD**Y**SEADGLSER (68)	−3.678	0.021	May confer stability and plasticity to neuronal membrane	Unknown
Band 4.1‐like protein 3	Epb41l3, Q9WV92	DSVSAAEVGTGQ**Y**ATTK (479)	−2.577	0.024	Inhibits cell proliferation and promotes apoptosis.	Human isoform 2 (Y471) controlled by ephrin_B1.
Calcium‐dependent secretion activator 2	Cadps2, Q8BYR5	T**Y**DTLHR (1273)	−3.236	0.012	Exocytosis	Novel phosphorylation site
Calmodulin‐regulated spectrin‐associated protein 2	Camsap1 l1, Q8C1B1	EEAAGAEDEKV**Y**TDR (799)	−2.566	0.032	Regulator of noncentrosomal microtubule dynamics and organization.	Novel phosphorylation site
Calmodulin‐regulated spectrin‐associated protein 3	Kiaa1543, Q80VC9	API**Y**ISHPENPSK (520)	−2.143	0.033	Regulator of noncentrosomal microtubule dynamics and organization.	Unknown
Casein kinase I isoform delta	Csnk1d, Q9DC28	**Y**ASINTHLGIEQSR (179)	−2.342	0.012	Cellular component morphogenesis and endocytosis	Novel phosphorylation site
CLIP‐associating protein 2	Clasp2, Q8BRT1	DYNP**Y**NYSDSISPFNK (1014)	−2.972	0.044	Cell cycle.	Unknown
CMRF35‐like molecule 8	Cd300a, Q6SJQ0	AE**Y**SEIQKPR (303)	−4.043	0.048	Intracellular protein transport and receptor‐mediated endocytosis	Unknown
Coronin‐1A	Coro1a, O89053	ADQC**Y**EDVR (25)	−3.084	0.038	Cytoskeleton organization	Unknown
		HVFGQPAKADQC**Y**EDVR (25)	−5.976	0.042		
Coronin‐2A	Coro2a, Q8C0P5	ENC**Y**DSVPITR (26)	−2.633	0.040	Cytoskeleton organization	Unknown
Cytochrome c‐type heme lyase	Hccs, P53702	AYD**Y**VECPVTGAR (67)	−5.111	0.006	Cell cycle and coenzyme metabolic process	Unknown
Cytoplasmic dynein 1 heavy chain 1	Dync1h1, Q9JHU4	AISKDHL**Y**GTLDPNTR (2263)	−4.501	0.031	Cellular component morphogenesis, Cell cycle, Cellular component movement, Intracellular protein transport	Unknown
Dynamin‐1	Dnm1, P39053	RIEGSGDQIDT**Y**ELSGGAR (354)	−2.404	0.044	Cellular component morphogenesis, Intracellular protein transport, Endocytosis	Unknown
		IEGSGDQIDT**Y**ELSGGAR (354)	−2.893	0.045		
		EVDE**Y**KNFRPDDPAR (314)	−3.247	0.018		Novel phosphorylation site
		LQSQLLSIEKEVDE**Y**K (314)	−4.276	0.038		
Dynamin‐1‐like protein	Dnm1l, Q8K1M6	NKL**Y**TDFDEIR (107)	−2.832	0.047	Cellular component morphogenesis, Intracellular protein transport, Endocytosis	Novel phosphorylation site
EH domain‐containing protein 3	Ehd3, Q9QXY6	ELVNNLAEI**Y**GR (339)	−3.179	0.043	Synaptic transmission, Intracellular protein transport, Endocytosis, Neurotransmitter secretion	Unknown
Eukaryotic translation initiation factor 4 gamma 1	Eif4g1, Q6NZJ6	KVE**Y**TLGEESEAPGQR (1424)	−2.610	0.029	Apoptosis	Unknown
Glutaminase kidney isoform	Gls, D3Z7P3	YAIAVNDLGTE**Y**VHR (309)	−5.463	<0.001	Cellular amino acid biosynthetic catabolic process	Unknown
Glutathione S‐transferase P 1	Gstp1, P19157	YVTLIYTNYENGKND**Y**VK (119)	−2.652	0.029	Conjugation of reduced glutathione	Novel phosphorylation site
Heat shock protein 105	Hsph1, Q61699	NAVEECV**Y**EFRDK (644)	−2.354	0.043	Protein complex assembly	Unknown
LysM and putative peptidoglycan‐binding domain‐containing protein 2	Lysmd2, Q9D7V2	DEESP**Y**AASLYHS (208)	−3.571	0.030	Unknown	Unknown
Microtubule‐associated protein 6	Map6, Q7TSJ2	SL**Y**SEPFKECPK (493)	−3.469	0.020	Microtubule stabilization	Unknown
Myosin‐Va	Myo5a, Q99104	RTDSTHSSNESE**Y**TFSSEFAETEDIAPR (1124)	−6.827	0.041	Cellular component morphogenesis, Intracellular signal transduction, Cell cycle, Cytokinesis, Muscle development, Intracellular protein transport, Muscle contraction, Sensory perception	Unknown
Myotrophin	Mtpn, P62774	D**Y**VAKGEDVNR (21)	−3.285	0.005	Promotes dimerization of NF‐kappa‐B subunits and regulates NF‐kappa‐B transcription factor activity	Unknown
Myotubularin‐related protein 5	Sbf1, Q6ZPE2	RSTSTL**Y**SQFQTAESENR (1751)	−4.030	0.005	Intracellular protein transport, Phospholipid metabolic process	Human analog controlled by Ephrin B1
Neurochondrin	Ncdn, Q9Z0E0	SMIDDT**Y**Q**C**LTAVAGTPR (156)	−3.042	0.044	Signal transduction	Novel phosphorylation site
Peptidyl‐prolyl cis‐trans isomerase A	Ppia, P17742	SI**Y**GEKFEDENFILK (79)	−2.432	0.049	Accelerate the folding of proteins	Unknown
Phosphatidylethanolamine‐binding protein 1	Pebp1, P70296	L**Y**EQLSGK (181)	−2.263	0.040	Binds ATP, opioids and phosphatidylethanolamine	Regulated by Catsper1 in murine sperm
Phosphoglycerate kinase 1	Pgk1, P09411	LGDV**Y**VNDAFGTAHR (161)	−3.703	0.011	Glycolysis	Unknown
Probable cationic amino acid transporter	Slc7a14, Q8BXR1	EQALHQST**Y**QR (693)	−2.369	0.040	Amino acid transporter, Anion transport	Unknown
Probable G‐protein‐coupled receptor 158	Gpr158, Q8C419	KL**Y**AQLEIYKR (722)	−2.507	0.003	G‐protein‐coupled receptor signaling	Novel phosphorylation site
Programmed cell death 6‐interacting protein	Pdcd6ip, Q9WU78	I**Y**GGLTSK (608)	−4.124	0.002	Unknown orphan receptor.	Novel phosphorylation site
Protein arginine N‐methyltransferase 8	Prmt8, Q6PAK3	RGEEI**Y**GTISMKPNAK (355)	−2.181	0.039	Chromatin organization, Regulation of nucleobase‐containing compound metabolic process, Biosynthetic process, Transcription	Unknown
Protein EFR3 homolog B	Efr3b, Q6ZQ18	KKEAP**Y**MLPEDVFVEKPR (629)	−2.364	0.006	Component of a complex required to localize phosphatidylinositol 4‐kinase (PI4K) to the plasma membrane	Novel phosphorylation site
Protein FAM126B	Fam126b, Q8C729	**Y**STISLQEDR (487)	−2.301	0.026	Mediates cellular transport and reorganization of the microtubule cytoskeleton	Unknown
Protein kinase C and casein kinase substrate in neurons protein 1	Pacsin1, Q61644	GPQ**Y**GSLER (74)	−3.649	0.012	Cellular component morphogenesis, Cell differentiation, Nervous system development, Endocytosis	Novel phosphorylation site
Protein XRP2	Rp2, Q9EPK2	D**Y**MFSGLKDETVGRLPGK (37)	−3.700	0.025	Purine nucleobase metabolic process	Unknown
Putative tyrosine‐protein phosphatase auxilin	Dnajc6, Q80TZ3	HLDHYTV**Y**NLSPK (134)	−2.616	0.034	Intracellular protein transport, Endocytosis	Novel phosphorylation site
Receptor‐type tyrosine‐protein phosphatase‐like N	Ptprn, Q60673	LAALGPEGAHGDTTFE**Y**QDLCR (628)	−2.592	0.031	Plays a role in vesicle‐mediated secretory processes	Unknown
Regulator of G‐protein signaling 6	Rgs6, Q9Z2H2	SV**Y**GVTDETQSQSPVHIPSQPIRK (234)	−3.918	0.042	Regulation of nucleobase‐containing compound metabolic process, Regulation of phosphate metabolic process, Catabolic process	Novel phosphorylation site
Rho GTPase‐activating protein 1	Arhgap1, Q5FWK3	HQIVEVAGDDK**Y**GR (81)	−2.031	0.043	Regulation of nucleobase‐containing compound metabolic process, Regulation of phosphate metabolic process, Regulation of catalytic activity, Catabolic process.	Unknown
Rho GTPase‐activating protein 35	Arhgap35, Q91YM2	KMQASPEYQD**Y**VYLEGTQK (308)	−3.163	0.035	Regulation of nucleobase‐containing compound metabolic process, Regulation of phosphate metabolic process, Regulation of catalytic activity, Catabolic process	Growth factors induce phosphorylation of thyrosine at position 308, which disrupts its ability to bind with General Transcription Factor II‐I.
SH3 and cysteine‐rich domain‐containing protein 2	Stac2, Q8R1B0	ESPPTGTSGKVDPV**Y**ETLR (205)	−2.054	0.044	Unknown	Unknown
SH3 and PX domain‐containing protein 2B	Sh3pxd2b, A2AAY5	TEPAQSEDHVDI**Y**NLR (661)	−3.128	0.040	Intracellular signal transduction	Unknown
SLIT‐ROBO Rho GTPase‐activating protein 3	Srgap3, Q812A2	NDLQSPTEHISD**Y**GFGGVMGR (845)	−2.312	0.024	Regulation of nucleobase‐containing compound metabolic process, Regulation of phosphate metabolic process, Cellular component movement, Locomotion, Catabolic process	Unknown
Src substrate cortactin	Cttn, Q60598	NASTFEEVVQVPSA**Y**QK (334)	−3.605	0.030	Cellular component morphogenesis	Phosphorylation is by proto‐oncogene tyrosine‐protein kinase Src and dephosphorylation is by protein tyrosine phosphophatase 1B.
Synaptic vesicle glycoprotein 2B	Sv2b, Q8BG39	YRDN**Y**EGYAPSDGYYR (10)	−2.635	0.004	Synaptic transmission, Neurotransmitter secretion	Unknown
		DN**Y**EGYAPSDGYYR (10)	−3.735	0.016		
Synaptogyrin‐3	Syngr3, Q8R191	G**Y**QVPAY (224)	−3.971	0.026	Positive regulation of dopamine transporter activity	Novel phosphorylation site
Syntaxin‐binding protein 1	Stxbp1, O08599	ERISEQT**Y**QLSR (473)	−2.563	0.035	Lysosomal transport, Intracellular protein transport, Synaptic vesicle exocytosis, Neurotransmitter secretion	Unknown
		ISEQT**Y**QLSR (473)	−2.843	0.015		
		**Y**STHLHLAEDCMK (344)	−2.920	0.019		Novel phosphorylation site
		H**Y**QGTVDKLCR (358)	−3.161	0.025		Novel phosphorylation site
Thioredoxin reductase 1, cytoplasmic	Txnrd1, Q9JMH6	VVYENA**Y**GR (245)	−2.016	0.032	Respiratory electron transport chain	Phosphorylation is by proto‐oncogene tyrosine‐protein kinase Src. Phosphorylation of human analog (tyrosine 281) reduced by ZAP70 and upregulated in neuroblastoma.
Triosephosphate isomerase	Tpi1, P17751	II**Y**GGSVTGATCK (259)	−2.893	0.029	Glycolysis and gluconeogensis	Unknown
Tubulin polymerization‐promoting protein	Tppp, Q7TQD2	VDLVDESG**Y**VPGYK (200)	−3.438	0.008	Microtubule functions	Unknown
Type I inositol 3,4‐bisphosphate 4‐phosphatase	Inpp4a, Q9EPW0	VQDDGGSDQN**Y**DVVTIGAPAAHCQGFK (355)	−2.552	0.013	Regulation of megakaryocyte and fibroblast proliferation	Unknown
		HYRPPEGT**Y**GKVET (934)	−3.111	0.031		Unknown
Tyrosine‐protein kinase	Lyn, P25911	VIEDNE**Y**TAR (397)	−2.289	0.015	Cell adhesion, Transmembrane receptor protein tyrosine kinase signaling, Cell proliferation, Cellular component movement, Cell differentiation, Apoptosis, Hemopoiesis, Nervous system development, Immune system response, Exocytosis, Locomotion, Coagulation, Stress response	Phosphorylation of tyrosine in at tyrosine 397, located within the activation loop, is required for its kinase activity. Lyn is activated by B‐cell receptor and inhibited by CD45.
UPF0554 protein C2orf43 homolog	Ldah, Q8BVA5	IEDV**Y**GLNGQIEHK (111)	−2.475	0.015	Serine lipid hydrolase associated with lipid droplets	Novel phosphorylation site
Vesicular inhibitory amino acid transporter	Slc32a1, O35633	SEGEPCGDEGAEAPVEGDIH**Y**QR (Y85)	−2.321	0.020	Amino acid transporter, Anion transport	Unknown
Wolframin	Wfs1, P56695	N**Y**IALDDFVELTKK (242)	−7.251	0.045	Regulation of cellular Ca2 + homeostasis	Unknown

a Underlined amino acid represents phosphorylated tyrosine and numbers in brackets indicate the phosphotyrosine position.

## Discussion

Our study has demonstrated that CN‐105, an apoE‐mimetic pentapeptide, reduces mortality, infarct volume, microgliosis, and improves long‐term functional outcome in a murine reperfusion model of ischemic stroke. We have previously demonstrated that larger peptides derived from the apoE receptor‐binding region (apoE133‐149) retain the neuroprotective and anti‐inflammatory properties^8^, ^17^, ^30^, ^52^, ^53^ of the apoE holoprotein. However, these peptides were limited by their relatively larger size, resulting in lower CNS penetration and higher cost of production. CN‐105, our current apoE‐mimetic pentapeptide, retains the beneficial neuroprotective effect in ischemic stroke observed in other apoE‐mimetic peptides^31^, ^32^ and possesses increased CNS penetration and potency.^35^

**Figure 7:**
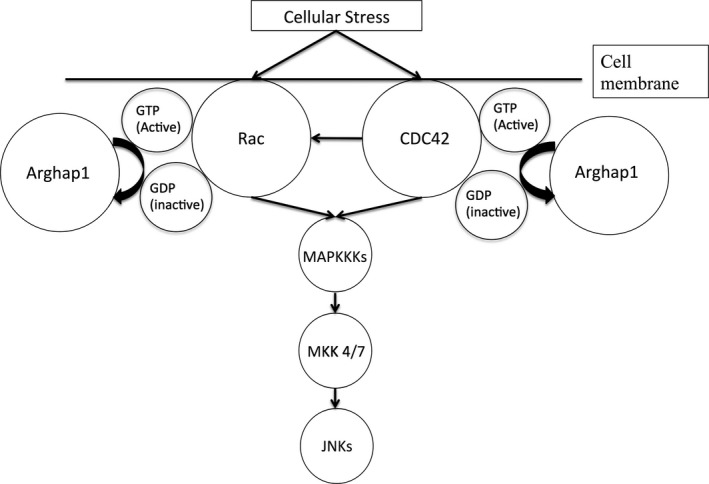
Rho GTPase‐activating protein 1 (Arghap1) downregulates c‐JNKs pathway through inactivation of stress‐induced proteins. External cellular stress induces activation of Ras‐Related C3 Botulinum Toxin Substrate (Rac) and Cell Division Cycle‐42 (CDC42). This triggers the sequential activation of mitogen‐activated protein kinase kinase kinases (MAPKKKs) and mitogen‐activated protein kinase kinases 4/7, resulting in JNKs activation. Arghap1 accelerate the intrinsic GTPase activity of Rac and CDC42 to return it to the inactive GDP‐bound conformation, resulting in downregulation of subsequent pathways. JNKs, Jun N‐terminal kinases.

We demonstrated that CN‐105 downregulates microglial activation in ischemic stroke and suppress TNF‐*α* secretion from C8‐B4 microglial cells. These immune‐modulatory effects on microglial by CN‐105 are consistent with apoE^15^, ^16^, ^17^, ^54^ and other apoE‐mimetic peptides.^17^, ^34^ LRP1, a cell surface receptor, has been shown to bind apoE^55^ and other apoE‐mimetic peptides.^29^, ^56^ The function of this interaction between apoE and LRP1, has been associated with suppression of neuroinflammation.^57^ Microglial cells, which expresses LRP1, are resident macrophages within the CNS that become activated in pathological situations, including cerebral ischemia.^58^ Microglial activation results in oxidative stress and the release of inflammatory mediators, contributing to BBB breakdown, secondary neuronal injury, and development of cerebral edema.^59^ There is evidence that apoE‐mimetic peptides reduce these inflammatory responses in microglial cells, via LRP1, through the c‐Jun N‐terminal kinases (JNKs) pathway.^60^, ^61^ JNKs are stress‐activated protein kinases that mediate the activation of microglia.^62^ External cellular stress, such as ischemia, induces a complex system of upstream signals, which in turn activates JNKs and results in expression of transcription factors, such as c‐Jun or c‐Fos, that participate in apoptosis and many cell death paradigms.^62^ Our early phosphoproteomic results support the hypothesis that CN‐105 mediates the downregulation of JNK pathway, through the identification of the differentially phosphorylated Rho GTPase‐activating protein 1 (Arghap1). Arghap1 serves as a upstream molecular switch which converts various stress‐induced proteins into an inactive state^63^ (Fig. 7). These inactivated proteins are then unable to transmit signals to activate JNKs downstream.^64^

Our study revealed a novel finding of increased microglial activation in the uninjured contralateral hippocampus after experimental ischemic stroke. Although the blood supply is not interrupted, it has been previously reported that the “healthy” contralateral hemisphere of an ischemic brain is associated with radiographic changes^65^ in the immediate poststroke period. Secretion of inflammatory mediators and trophic factors by non‐neuronal cells^66^ has been implicated. Our current finding of elevated microglial cell density in the contralateral hippocampus suggest that global and potentially detrimental inflammatory changes exist throughout the brain following focal ischemia. Remote downregulation of microglial activation in the contralateral uninjured hippocampus by CN‐105 suggests its anti‐inflammatory benefit extends beyond the effect of reducing the ipsilateral infarct volume.

A possible mechanism by which CN‐105 exerts its anti‐inflammatory and neuroprotective effect may be through the preservation of the BBB. After ischemic stroke, BBB disruption occurs and persists for days. ApoE appears to protect cerebrovascular integrity in acute brain injury through maintenance of the BBB, as demonstrated in apoE‐deficient mice, which was associated with an increase in disruption of BBB after injury.^67^, ^68^, ^69^ More recently, it has been found that apoE stabilizes BBB through suppression of cyclophilin A (Peptidylprolyl cis‐trans isomerase A or CypA) in an isoform‐specific manner via interaction with LRP1.^57^ We observed, through our phosphoproteomic study, that CN‐105 downregulated phosphorylation of CypA in ischemic stroke. This is consistent with the hypothesis that CN‐105 downregulates CypA and results in improved BBB integrity following ischemic stroke. Additionally, the phosphorylation site identified at tyrosine 79 of CypA in our data possibly points toward a critical signaling mechanism between CypA and either the upstream LRP1 receptor or downstream nuclear‐factor‐keppa‐beta transcription factor^61^ and deserves further investigation. CypA has also been additionally implicated in posthypoxic apoptosis via apoptosis‐inducing factor,^70^ suggesting that downregulation of CypA by CN‐105 may confer other neuroprotective effects.

Reduction in early mortality (24–72 h) after ischemic stroke was observed in our study with CN‐105. Early mortality in acute ischemic stroke is associated with initial clinical stroke severity, the size of the infarct and subsequent cerebral edema.^71^, ^72^, ^73^ Mechanism which CN‐105 reduced early mortality were likely through decreasing overall infarct size, as observed in our study, and also through decreasing cerebral edema by maintaining BBB integrity. Inflammation begins within hours in the postischemic brain,^74^, ^75^ resulting in activation of microglial and secretion of inflammatory cytokines within 24 h of infarction.^76^ These inflammatory cytokines contributes to cerebral edema through disruption of BBB integrity.^77^ Additionally, through the downregulation of phophorylation of CypA, as demonstrated in our phosphoproteomic results, CN‐105 also maintains BBB integrity, reducing cerebral edema after injury. All these factors contributes to the reduction in early mortality.

Other differentially phosphorylated proteins may also provide clue toward CN‐105′s mechanism of action. For example, CN‐105 may suppress microglial activation through the deactivation of Lyn. Lyn belongs to the Src family of tyrosine kinases predominantly expressed in granulocytes^78^ and involved in early B‐cell signaling pathway.^79^ Inhibition of Lyn showed suppression of microglial activation in an Alzheimer's disease model.^80^ Phosphorylation at tyrosine 397 (p‐Tyr397‐Lyn) is required for Lyn to perform its functional kinase activity^81^ to effect downstream proinflammatory pathways.^79^ Hence our observation of downregulation of p‐Tyr397‐Lyn suggests a possible mechanism by which CN‐105 reduces microglial activation. Moreover, Lyn activation has been implicated in degranulation of mast cells^82^ releasing histamine, consistent with our previous observation that apoE‐deficient mice possess elevated histamine after acute brain injury.^83^

Our study was limited by the inability to demonstrate improved functional outcome when CN‐105 was administered at 0.1 mg/kg beyond a latency of 30 min from ischemia. Although the protective effect of CN‐105 appears to be time‐dependent, our study was focused on delineating the early mechanistic effects of CN‐105 in ischemic stroke, and did not test the effects of higher drug dosage or multiple dosing, which have increased efficacy in prior studies.^32^ Since CN‐105 does not include the polymorphic region of the apoE holoprotein, the differential effects of ApoE isoforms were not investigated. An inherent limitation of the phosphoproteomic approach was the use of a twofold cutoff to determine significance between treatment groups. This could have excluded certain important phosphopeptides, with small initial changes, that may be associated with amplified downstream neuroprotective effects. The efficacy of CN‐105 would also have been further enhanced if evaluated in different models of ischemic stroke and female mice. Additionally, to elucidate if CN‐105 works definitively through LRP1, antagonism or genetic knock‐out models of LRP1 will be needed.

In summary, we demonstrate that intravenous administration of CN‐105 conferred durable sensorimotor functional improvements, reduced mortality, histological inflammation, and inflammatory phosphoproteomic signals following cerebral ischemia. In vitro application of CN‐105 also reduced microglial activation. With safety already demonstrated in a clinical trial, CN‐105 may be an attractive candidate for further clinical evaluation in ischemic stroke.

## Author Contributions

T.M.T. designed the study, performed the in vivo experiments and functional testing, analyzed the data, and wrote the manuscript. B.J.K. designed the study and wrote the manuscript. E.J.S designed, collected and analyzed data, and wrote the manuscript. V.C. contributed to the in vivo experiments, immunohistochemistry assessments and wrote the manuscript. P.F. performed the in vitro experiments and wrote the manuscript. H.W. designed the study and contributed to the animal experiments. M.A.M. had scientific oversight of the proteomic component. H.N.D. assisted the study design, immunohistochemistry assessment, and edited the manuscript. D.T.L. designed the study, acquired grant, supervised the work, and wrote the manuscript. All authors reviewed the full manuscript.

## Conflict of Interest

D.T.L, B.J.K, and H.N.D are co‐inventors of CN‐105 with US patent 9,303,063. D.T.L serves as an officer for Aegis‐CN, LLC. Aegis‐CN, LLC supplied the drug, but had no editorial control over study design, conduct, or writing of the manuscript.

## Supporting information


**Data S1.** Physiological studies of CN‐105.

**Data S2.** Supplemental methods.

**Data S3.** Supplemental results.

**Table S1.** Principal biological process of differentially significant phosphoproteins.

**Table S2.** Known cell signaling pathways of differentially expressed phosphoproteins.

**Table S3.** Biological functions of phosphoproteins that are significantly different between CN‐105 and vehicle in ischemic stroke.
