# Supplementary material for: Apolipoprotein E mimetic peptide, CN‐105, improves outcomes in ischemic stroke
Source: Ann Clin Transl Neurol. 2017 Mar 9;4(4):246–65. doi: 10.1002/acn3.399 (PMC5376751; doi:10.1002/acn3.399)
Supplement: Supplementary file 1 — Data S1. Physiological studies of CN‐105. [file ACN3-4-246-s001.pdf]

Supplementary Material 1: Physiological experiments of CN-105

Materials and methods

Vehicle and Test Article Preparation

The vehicle, 0.9% Sodium Chloride for Injection, USP, was prepared prior to handling the test article, by filtering an appropriate volume under a laminar flow hood using sterile equipment and aseptic techniques through a 0.22 micron polyvinylidene difluoride (PVDF) filter into a sterile receiver. Filtered vehicle was stored refrigerated at 2 to 8°C.

The test article, CN-105, was mixed with vehicle for at least 5 minutes using a magnetic stir bar and stir plate and until a visually clear, particulate free mixture was obtained. The contents of the mixing container were quantitatively transferred into a graduated cylinder, and the formulation was brought to volume with additional vehicle to achieve the final concentration of 1.25 mg/mL. The contents of the graduated cylinder were mixed thoroughly by inversion. The formulation was returned to the mixing container and stirred for at least 5 minutes using a magnetic stir bar and stir plate and until uniform in appearance. Under a laminar flow hood using sterile equipment and aseptic techniques, the formulation was filtered through 0.22 micron PVDF filter unit into an appropriate number of sterile receivers. Formulations of the test article were stored refrigerated at 2 to 8°C.

Animal Acquisition and Acclimation

Twenty male and 20 female experimentally naïve CD [Crl:CD(SD)] rats of approximately 8 weeks of age at receipt (Charles River Laboratories, Portage, Michigan) were used. All animals were received with a previously implanted femoral vein catheter exteriorized between the scapulae for intravenous (IV) dosing purposes. During the acclimation period, the animals were observed daily with respect to general health and any signs of disease. All animals were given a detailed clinical examination prior to selection for study.

Randomization, Assignment to Study, and Maintenance

Animals were assigned to the control and treatment groups via randomization. Each animal was assigned an animal number to be used in the Provantis data collection system and was implanted with a microchip bearing a unique identification number. The individual animal number, implant number, and study number comprised a unique identification for each animal. Each cage was identified by the animal number, study number, group number, and sex. The animals were individually housed in solid bottom cages with nonaromatic bedding in an environmentally controlled room. Fluorescent lighting was provided for approximately 12 hours per day. The dark cycle was interrupted intermittently due to study-related activities. Temperature and humidity were continuously monitored, recorded, and maintained to the maximum extent possible within the ranges of 68 to 79°F and 30 to 70%, respectively. Block Lab Diet (Certified Rodent Diet #5002, PMI Nutrition International, Inc.) was available ad libitum, except during designated periods. Tap water was available ad libitum to the additional animals received via

water bottles at receipt and throughout the duration of the study.

Drug/Vehicle administration

The vehicle (0 mg/kg/day) and test article (20 mg/kg/day) were administered intravenously (IV) via the surgically implanted femoral catheter. The test article and vehicle were administered over a 5 minute period four times per day for 14 consecutive days, 6 hours apart (±30 minutes from the start of one dose to start of next dose), for a total of 56 doses per animal. The total daily dose was split equally between the four daily doses.

Prior to and between each dose, the animals received a continuous infusion of 0.9% Sodium Chloride for Injection, USP, (saline) at a rate of approximately 0.4 mL/hr in order to maintain patency of the IV catheter. On the days of dosing, the infusion line was pre-filled with the dosing solution to ensure that dosing started as soon as the infusion pump was turned on. To ensure delivery of the complete dose, any dosing formulation remaining in the infusion line was flushed with saline administered at the same rate used for the test article (i.e., 48 mL/kg/hr) and was administered as part of the 5-minute infusion period. The dosing pumps were paused for approximately 1 minute between the dose and the flush to switch out syringes. Following the flush period, the infusion rate (saline) was returned to approximately 0.4 mL/hr.

Cageside Observations

All animals were observed for mortality and the availability of food and water twice daily. On

dosing days, the daily observations were conducted at 30 minutes post the end of the dose.

Body weight measurements

Body weights for all animals were measured and recorded prior to randomization, and weekly during the study. The body weights collected prior to randomization are reported at the Week -1 interval.

Respiratory Monitoring

Respiratory evaluations were performed on the first eight main study male animals per group. Animals were placed in a jacket and tether system prior to placement into the monitoring chamber. Each designated animal was placed in a plethysmograph chamber at least 2.5 hours prior to dosing. After at least 1.5 hours, respiratory monitoring was initiated to establish baseline data. The animals were dosed after at least 1 hour of continuous baseline recording and respiratory monitoring was continued for a period of at least 4 hours following the start of the first administration (Dose 1).

Data were logged into 1-minute intervals and reported in 15-minute time intervals. Only 1 hour of baseline data were reported. Food and water were not available to the animals during respiratory recording sessions. Respiratory rate, tidal volume and minute volume were monitored.

Blood analysis

Blood analysis was conducted on all main study animals at the terminal necropsy. The animals had access to drinking water but were fasted overnight prior to scheduled sample collection. Blood samples (approximately 4 mL) were collected via the vena cava after carbon dioxide inhalation. The samples were collected into tubes containing K_3EDTA for evaluation of hematology parameters, sodium citrate for evaluation of coagulation parameters, and serum separators with no anticoagulant for the clinical chemistry samples. The order of bleeding was by alternating one animal from each dose group, then repeating to reduce handling and time biases.

Statistical analysis

For all hematological, biochemistry, body weight and temperature endpoints where sample sizes for all groups were three or greater, Levene's test was used to assess homogeneity of group variances. If Levene's test was significant ($p<0.01$), comparisons with the control group were made using Welch's t-test with a Bonferroni correction. If Levene's test was not significant ($p\geq0.01$), a pooled estimate of the variance (Mean Square Error or MSE) was computed from a one-way analysis of variance (ANOVA) and utilized by a Dunnett's comparison of each treatment group with the control group; however, because there were only two groups involved, the above methodology for testing homogeneity of variance applied and the Dunnett's test was reduced to a Student's t-test. When there was no variability, no inferential statistics were done. Results of all pair-wise comparisons are reported at the 0.05 and 0.01 significance levels. All endpoints were analyzed using two-tailed tests.

For all respiratory end-points (respiratory rate and minute volume), descriptive statistics and repeated measures analysis of covariance were used. The data were tabulated within each summary time interval and the arithmetic mean (Mean), number of subjects (N), least squares mean (LSMean), and standard error of the LSMean (LSMean s.e.) were calculated for each endpoint and treatment. The effect of treatment over time was evaluated using a repeated measures analysis ofcovariance. Factors in the model included treatment, time (after dose), the two-way interaction 'treatment by time', and covariate (average of the 1 hour of predose data). A mixed model approach was used for the analysis using an appropriate covariance structure. Results from the model with the smallest Akaike's Information Criterion (AIC) were used. The first order Kenward-Roger (KR) degrees of freedom approximation was utilized. Figures representing the LS Means were generated for each respiratory endpoint to supplement the statistical evaluation of response to treatment.

Monotonicity of dose response was examined using sequential linear trend tests based on ordinal spacing of dose levels. Two linear 'trend by time' interaction tests were performed: 1) high-dose 'linear trend by linear time' and 2) high-dose 'linear trend by quadratic time', both evaluated at the 0.05 significance level. Neither interaction was significant; therefore trend tests on treatment means were performed at the 0.05 level for the overall segmentmeans only. No significant linear trends were detected among the overall segment treatment means; therefore non-monotonic dose responses were investigated. The overall effects of treatment and the 'treatment by time' interaction were tested using overall F-tests at the 0.05 significance level. Neither the 'treatment by time' interaction effect nor the treatment main effect was significant; therefore no further

analysis was conducted.

Results of physiological study

Mortality

Three study animals were found dead during the course of the study, including
2 control (out of 20) animals (one male and one female) and 1 treated (out of 20) female. The animals died on Day 7, Day 6, and Day 9 of the study, respectively. Cause of death was undetermined for all animals. These deaths are not considered to be test article related due to the higher incidence in controls as compared to treated animals.

Body temperature

There was no difference in body temperature.

0 mg/kg/day			20 mg/kg/day		
Mean	SD	N	Mean	SD	N
Predose					
37.00	0.380	10	37.13	0.327	10
Immediate postdose					
36.70	0.249	10	36.70	0.521	10
4 hour postdose					
36.57	0.416	10	36.53	0.583	10

Body weight

Slight, decreases (5-6%) in mean weekly body weights, as compared to vehicle controls, were observed for both males and females, reaching statistical significance for females only. The observed decrease in body weight gain was likely related in part to lower body weights predose for both sexes, as compared to vehicle controls, despite randomization.

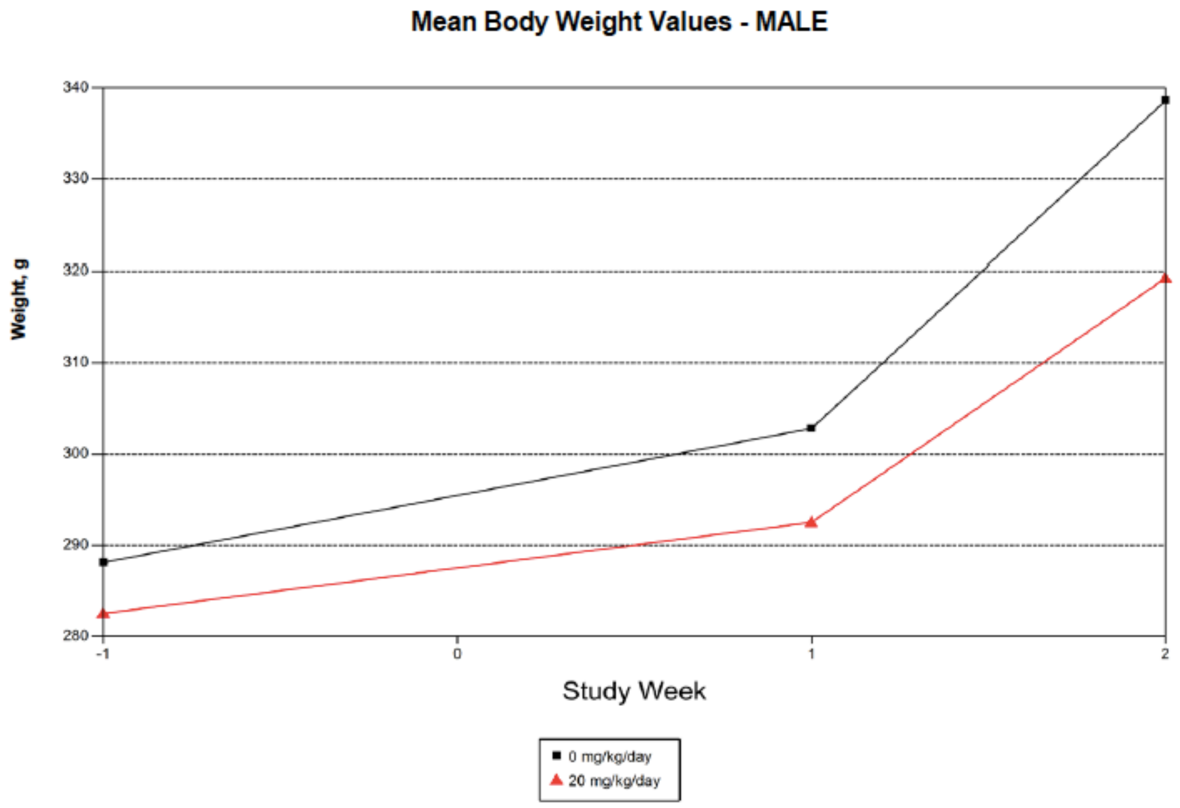

Summary of Body Weight Values - MALE

Endpoint	Study Interval (Week)	0 mg/kg/day Mean	SD	N	20 mg/kg/day Mean	SD	N
Body Weight g							
	-1	288.1	12.02	10	282.5	7.56	10
	1	302.8	19.20	10	292.5	16.00	10
	2	338.6	25.98	9	319.3	22.12	10

Mean Body Weight Values - FEMALE

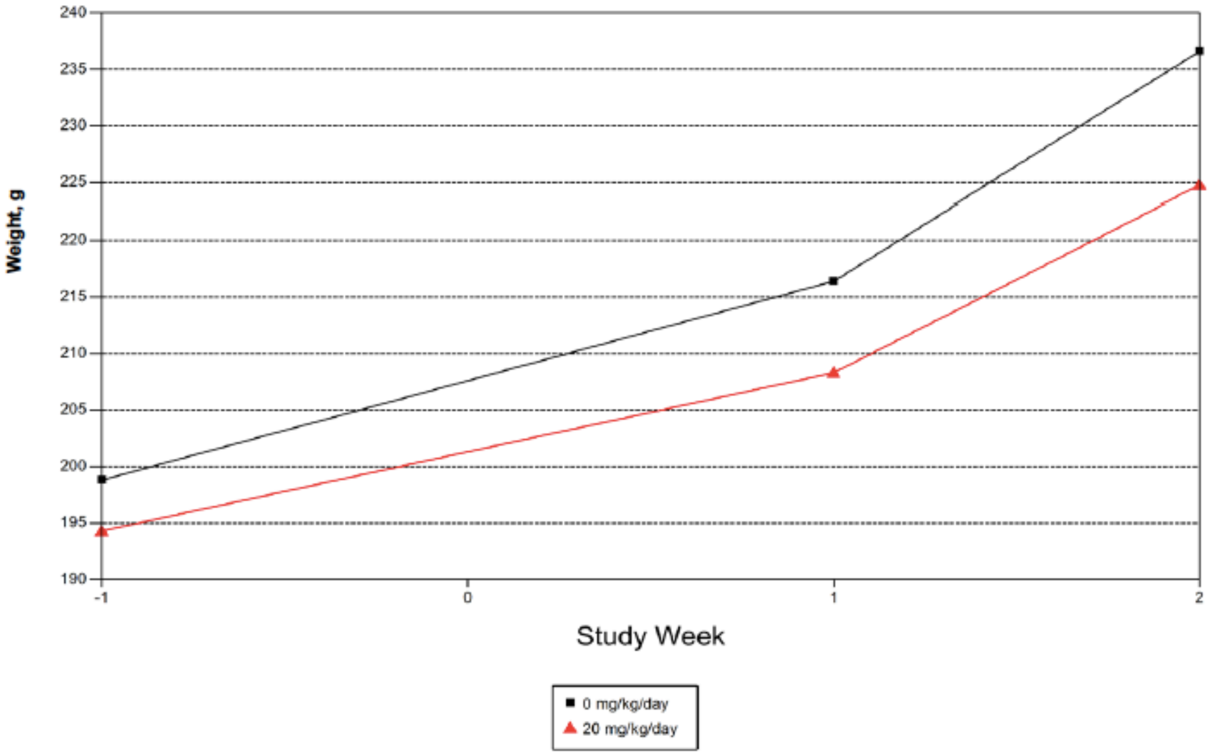

Summary of Body Weight Values - FEMALE

Endpoint	Study Interval (Week)	0 mg/kg/day Mean	SD	N	20 mg/kg/day Mean	SD	N
Body Weight g							
	-1	198.8	9.32	10	194.3	6.62	10
	1	216.4	9.00	10	208.3	12.87	10
	2	236.6	9.34	9	224.8[a]	13.53	9

[a] Significantly different from control; ($p<0.05$)

Respiratory Evaluations

LSMean respiratory rate values and minute volume values are illustrated and summarized below. There were no CN-105-related effects on respiratory function observed over the course of the study.

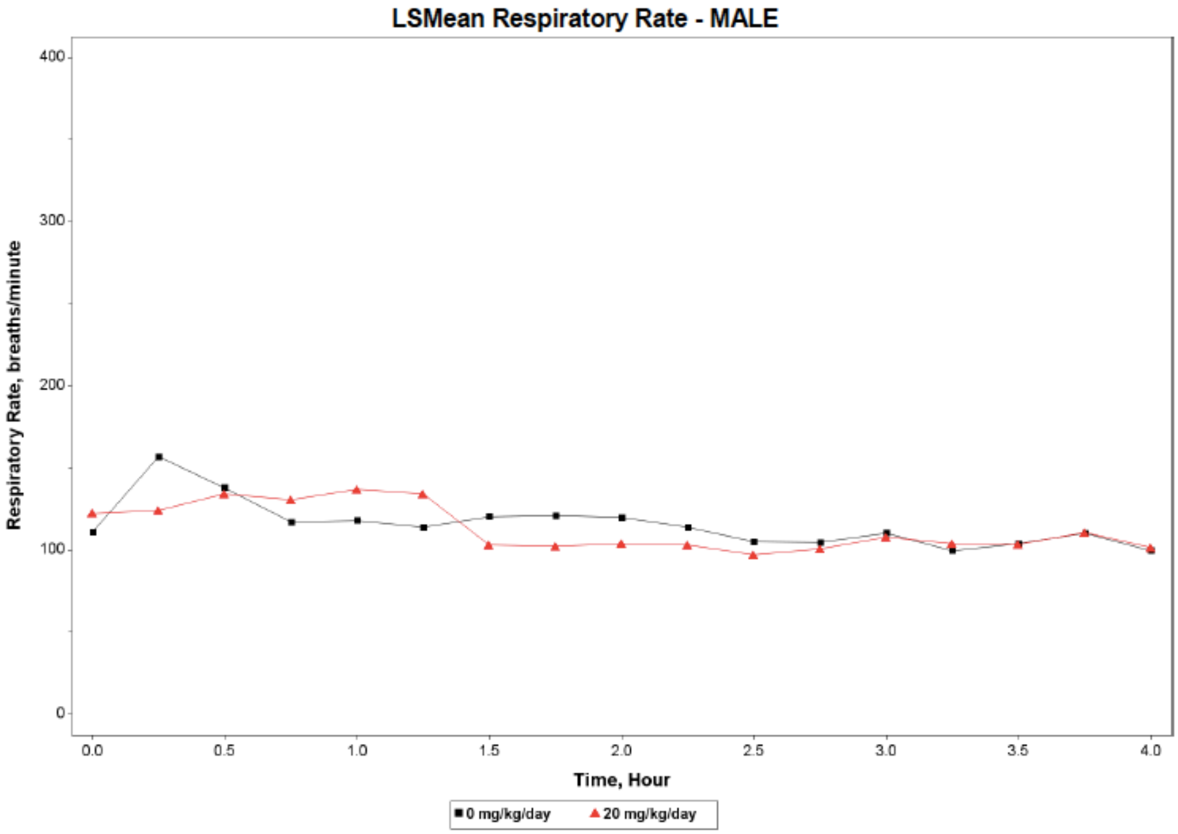

Summary of Respiratory Rate, breaths/minute - MALE
Analysis of 0:15 through 4:00 (hour:minute) Time Interval Values (Analysis Segment 1)
Covariate Mean = Average of 1-hour Predose, with ARH(1) Covariance Structure

Type		P-Value
TREND	Linear Group*Linear Time Trend	0.7806 (NS)
	Linear Group*Quadratic Time Trend	0.9036 (NS)
INTERACTION EFFECT	Group*Time	0.6787 (NS)
MAIN EFFECT	Group	0.5571 (NS)

Summary of Respiratory Rate, breaths/minute - MALE
Analysis of 0:15 through 4:00 (hour:minute) Time Interval Values (Analysis Segment 1)
Covariate Mean = Average of 1-hour Predose, with ARH(1) Covariance Structure

Group	Covariate Mean	Statistics	Overall	0:15	0:30	0:45	1:00	1:15	1:30	1:45	2:00	2:15
0 mg/kg/day	110.859	Mean	115.089	156.104	137.222	116.322	117.405	113.189	119.739	120.309	119.210	113.068
		N	8	8	8	8	8	8	8	8	8	8
		LS Mean	115.664	156.680	137.798	116.897	117.981	113.764	120.314	120.885	119.786	113.643
		LSM s.e.	3.885	18.343	16.832	11.385	14.928	14.721	8.880	11.211	7.824	4.875
20 mg/kg/day	122.395	Mean	112.929	124.915	134.601	131.039	137.544	134.897	103.494	102.932	104.260	103.748
		N	8	8	8	8	8	8	8	8	8	8
		LS Mean	112.353	124.339	134.025	130.463	136.968	134.321	102.919	102.357	103.684	103.173
		LSM s.e.	3.885	18.343	16.832	11.385	14.928	14.721	8.880	11.211	7.824	4.875
		Trend p-value	0.5571									

Summary of Respiratory Rate, breaths/minute - MALE
Analysis of 0:15 through 4:00 (hour:minute) Time Interval Values (Analysis Segment 1)
Covariate Mean = Average of 1-hour Predose, with ARH(1) Covariance Structure

Group	Covariate Mean	Statistics	2:30	2:45	3:00	3:15	3:30	3:45	4:00
0 mg/kg/day	110.859	Mean	104.408	104.047	109.675	98.979	103.283	109.478	98.981
		N	8	8	8	8	8	8	8
		LS Mean	104.984	104.623	110.251	99.555	103.858	110.054	99.557
		LSM s.e.	4.588	4.639	10.006	2.756	6.105	13.615	5.196
20 mg/kg/day	122.395	Mean	97.641	101.315	108.440	104.510	104.215	111.354	101.957
		N	8	8	8	8	8	8	8
		LS Mean	97.066	100.740	107.865	103.934	103.639	110.778	101.381
		LSM s.e.	4.588	4.639	10.006	2.756	6.105	13.615	5.196
		Trend p-value							

LSMean Minute Volume - MALE

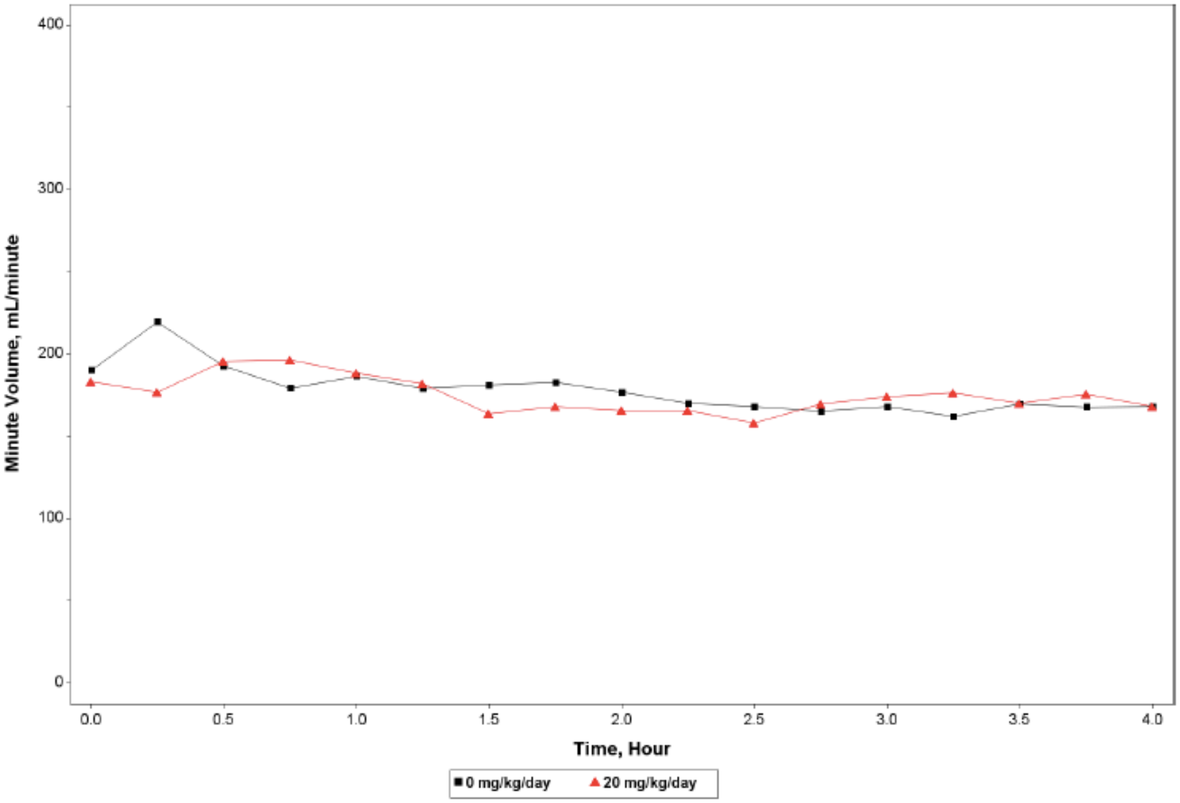

Summary of Minute Volume, mL/minute - MALE

Analysis of 0:15 through 4:00 (hour:minute) Time Interval Values (Analysis Segment 1)
Covariate Mean = Average of 1-hour Predose, with ARH(1) Covariance Structure

Type		P-Value
TREND	Linear Group*Linear Time Trend	0.2350 (NS)
	Linear Group*Quadratic Time Trend	0.8446 (NS)
INTERACTION EFFECT	Group*Time	0.4189 (NS)
MAIN EFFECT	Group	0.6360 (NS)

Summary of Minute Volume, mL/minute - MALE

Analysis of 0:15 through 4:00 (hour:minute) Time Interval Values (Analysis Segment 1)
Covariate Mean = Average of 1-hour Predose, with ARH(1) Covariance Structure

Group	Covariate Mean	Statistics	Overall	0:15	0:30	0:45	1:00	1:15	1:30	1:45	2:00	2:15
0 mg/kg/day	190.101	Mean	179.386	221.662	194.744	181.537	188.579	181.187	183.204	184.755	178.923	172.099
		N	8	8	8	8	8	8	8	8	8	8
		LS Mean	177.226	219.502	192.584	179.377	186.419	179.026	181.044	182.594	176.763	169.939
		LSM s.e.	3.811	14.317	14.177	11.897	14.268	10.188	11.516	11.871	5.912	4.391
20 mg/kg/day	183.223	Mean	172.412	174.633	193.304	194.144	186.106	179.760	161.658	165.887	163.379	163.656
		N	8	8	8	8	8	8	8	8	8	8
		LS Mean	174.572	176.793	195.464	196.304	188.266	181.920	163.818	168.047	165.539	165.817
		LSM s.e.	3.811	14.317	14.177	11.897	14.268	10.188	11.516	11.871	5.912	4.391
		Trend p-value	0.6360									

Summary of Minute Volume, mL/minute - MALE
Analysis of 0:15 through 4:00 (hour:minute) Time Interval Values (Analysis Segment 1)
Covariate Mean = Average of 1-hour Predose, with ARH(1) Covariance Structure

Group	Covariate Mean	Statistics	2:30	2:45	3:00	3:15	3:30	3:45	4:00
0 mg/kg/day	190.101	Mean	170.178	167.336	170.045	164.103	171.665	169.968	170.192
		N	8	8	8	8	8	8	8
		LS Mean	168.018	165.176	167.885	161.943	169.505	167.807	168.032
		LSM s.e.	5.507	6.591	9.156	4.891	7.548	13.418	5.682
20 mg/kg/day	183.223	Mean	155.809	167.449	171.695	174.084	167.920	173.284	165.820
		N	8	8	8	8	8	8	8
		LS Mean	157.969	169.609	173.856	176.244	170.081	175.445	167.980
		LSM s.e.	5.507	6.591	9.156	4.891	7.548	13.418	5.682
		Trend p-value							

Hematology and coagulation parameters

There were no test article-related effects among hematology parameters, coagulation times (i.e. APTT or prothrombin time) or fibrinogen in either sex administered 20 mg/kg/day at 14 days. All mean and individual values were within an acceptable range for biologic and procedure-related variation.

Summary of Hematology Values - MALE

Endpoint	Study Interval	0 mg/kg/day Mean	0 mg/kg/day SD	0 mg/kg/day N	20 mg/kg/day Mean	20 mg/kg/day SD	20 mg/kg/day N
Leukocytes $10^3/\mu L$	Terminal	12.90	3.151	9	12.52	3.402	10
Erythrocytes $10^6/\mu L$	Terminal	8.014	0.7955	9	8.192	0.6311	10
Hemoglobin g/dL	Terminal	15.64	1.702	9	15.68	1.434	10
Hematocrit %	Terminal	51.66	5.684	9	51.62	5.891	10
MCV fL	Terminal	64.50	4.424	9	62.90	3.253	10
MCH pg	Terminal	19.51	0.885	9	19.12	0.846	10
MCHC g/dL	Terminal	30.29	1.001	9	30.45	1.197	10
Platelets $10^3/\mu L$	Terminal	884.2	234.11	9	881.1	209.83	10
Absolute Reticulocytes $10^3/\mu L$	Terminal	245.97	52.756	9	253.34	51.560	10
Neutrophils $10^3/\mu L$	Terminal	2.197	0.6269	9	1.908	0.8581	10
Lymphocytes $10^3/\mu L$	Terminal	10.036	2.5480	9	9.955	2.8660	10
Monocytes $10^3/\mu L$	Terminal	0.266	0.1674	9	0.312	0.1841	10
Eosinophils $10^3/\mu L$	Terminal	0.177	0.0640	9	0.126[a]	0.0384	10
Basophils $10^3/\mu L$	Terminal	0.078	0.0721	9	0.104	0.1245	10
APTT sec	Terminal	16.18	1.339	8	15.19	2.271	8
Prothrombin Time sec	Terminal	16.05	0.849	8	16.46	0.981	9
Fibrinogen mg/dL	Terminal	458.1	74.31	8	506.7	91.39	9

Summary of Hematology Values - FEMALE

Endpoint	Study Interval	0 mg/kg/day			20 mg/kg/day		
		Mean	SD	N	Mean	SD	N
Leukocytes $10^3/\mu L$	Terminal	9.86	4.692	9	9.86	2.024	8
Erythrocytes $10^6/\mu L$	Terminal	7.779	0.4735	9	7.925	0.4186	8
Hemoglobin g/dL	Terminal	15.39	0.947	9	15.61	0.740	8
Hematocrit %	Terminal	50.32	3.789	9	51.23	2.839	8
MCV fL	Terminal	64.70	2.628	9	64.66	2.451	8
MCH pg	Terminal	19.77	0.464	9	19.71	0.708	8
MCHC g/dL	Terminal	30.59	1.265	9	30.54	1.041	8
Platelets $10^3/\mu L$	Terminal	914.9	203.02	9	901.1	142.08	8
Absolute Reticulocytes $10^3/\mu L$	Terminal	235.27	49.191	9	260.64	40.629	8
Neutrophils $10^3/\mu L$	Terminal	1.268	0.7349	9	1.259	0.6529	8
Lymphocytes $10^3/\mu L$	Terminal	8.102	3.9695	9	8.178	2.0508	8
Monocytes $10^3/\mu L$	Terminal	0.179	0.0655	9	0.161	0.0559	8
Eosinophils $10^3/\mu L$	Terminal	0.153	0.0941	9	0.121	0.0429	8
Basophils $10^3/\mu L$	Terminal	0.054	0.0224	9	0.050	0.0131	8
APTT sec	Terminal	13.51	1.667	9	14.05	1.818	8
Prothrombin Time sec	Terminal	15.68	0.360	9	15.61	0.704	8
Fibrinogen mg/dL	Terminal	371.7	76.83	9	347.1	74.50	8

Biochemistry parameters

Chloride was statistically increased (up to +2%) at the terminal collection in both sexes administered 20 mg/kg/day. Increases in chloride were not considered biologically relevant given the small magnitude of the changes. Despite a few other statistically significant alterations, no other test article-related effects were noted among clinical chemistry analytes in either sex administered 20 mg/kg/day. All other mean and individual values were within an acceptable range for biologic and procedure-related variation.

Summary of Clinical Chemistry Values - MALE

Endpoint	Study Interval	0 mg/kg/day Mean	SD	N	20 mg/kg/day Mean	SD	N
Sodium mEq/L	Terminal	142.7	1.94	9	142.4	2.27	10
Potassium mEq/L	Terminal	8.43	1.580	9	9.61	1.899	10
Chloride mEq/L	Terminal	100.6	1.24	9	102.4[a]	1.51	10
Calcium mg/dL	Terminal	11.42	0.474	9	11.36	0.392	10
Phosphorus mg/dL	Terminal	11.61	1.004	9	12.00	1.023	10
Alkaline Phosphatase U/L	Terminal	169.6	23.70	9	186.4	41.31	10
Total Bilirubin mg/dL	Terminal	0.11	0.033	9	0.14	0.052	10
AST U/L	Terminal	98.3	29.85	9	109.0	15.86	10
ALT U/L	Terminal	40.9	8.30	9	42.0	8.71	10
Urea Nitrogen mg/dL	Terminal	17.7	2.55	9	18.2	2.15	10
Creatinine mg/dL	Terminal	0.31	0.060	9	0.29	0.032	10
Total Protein g/dL	Terminal	5.99	0.226	9	6.01	0.318	10
Albumin g/dL	Terminal	2.94	0.181	9	2.77	0.279	10
Globulin g/dL	Terminal	3.04	0.279	9	3.24	0.398	10

[a] Significantly different from control; ($p<0.05$)

Summary of Clinical Chemistry Values - FEMALE

Endpoint	Study Interval	0 mg/kg/day			20 mg/kg/day		
		Mean	SD	N	Mean	SD	N
Sodium mEq/L	Terminal	139.8	3.38	9	140.0	1.32	9
Potassium mEq/L	Terminal	10.84	3.015	9	10.66	1.109	9
Chloride mEq/L	Terminal	101.6	1.01	9	102.9[b]	0.78	9
Calcium mg/dL	Terminal	11.90	0.497	9	11.56	0.482	9
Phosphorus mg/dL	Terminal	12.07	0.934	9	11.80	1.077	9
Alkaline Phosphatase U/L	Terminal	128.8	41.05	9	120.9	19.67	9
Total Bilirubin mg/dL	Terminal	0.10	0.000	9	0.12	0.044	9
AST U/L	Terminal	96.0	19.53	9	102.4	14.14	9
ALT U/L	Terminal	35.0	3.91	9	37.3	6.80	9
Urea Nitrogen mg/dL	Terminal	17.8	1.09	9	18.3	1.66	9
Creatinine mg/dL	Terminal	0.32	0.044	9	0.36	0.053	9
Total Protein g/dL	Terminal	6.49	0.257	9	6.38	0.367	9
Albumin g/dL	Terminal	3.33	0.312	9	3.26	0.240	9
Globulin g/dL	Terminal	3.16	0.279	9	3.12	0.282	9

[b] Significantly different from control; ($p<0.01$)
