# Supplementary material for: Apolipoprotein E mimetic peptide, CN‐105, improves outcomes in ischemic stroke
Source: Ann Clin Transl Neurol. 2017 Mar 9;4(4):246–65. doi: 10.1002/acn3.399 (PMC5376751; doi:10.1002/acn3.399)
Supplement: Supplementary file 2 — Data S2. Supplemental methods. [file ACN3-4-246-s002.pdf]

Supplemental Material 2: Supplemental Methods

Animal surgical details

Ten to 13 week old C57Bl/6J male mice (Jackson Laboratories, Bar Harbor, ME) were housed with a 12-hour light/dark cycle in standard acrylic cages and with ad libitum access to food and water. Mice were induced with 4.6% isoflurane, endotracheally intubated, and mechanically ventilated with 1.6% isoflurane in 30% O2/70% N2. A midline incision over the ventral surface of the neck was performed, the right common carotid artery was identified, and the right external carotid artery ligated and transected. Transient ligation of the right common carotid artery was performed to reduce perfusion and make the injury less variable. A silicone-coated 6-0 monofilament (Doccol Corp, Redlands, CA) was inserted into the proximal external carotid artery stump and advanced 11 mm into the internal carotid artery to occlude the middle cerebral artery for the MCAO groups. Rectal temperature was servo-regulated with surface heating/cooling at 37°C throughout the surgical procedure (YSI Inc, Yellow Springs, Ohio). Upon completion of the stipulated duration of ischemic occlusion, the monofilament was removed, right external carotid artery was closed and the right common carotid artery ligation was released. Skin incisions were closed with 4-0 silk sutures. Isoflurane was discontinued, and mice were extubated upon recovery of spontaneous respiration. Post-operatively, all mice received 0.5ml of 0.9% normal saline subcutaneous injection below the dorsum of the neck immediately post-operatively. Sham-operated mice underwent identical surgical procedures, including the transient occlusion of the right common carotid artery, the insertion of the microfilament but without advancement into the internal carotid artery, and underwent 45

minutes of isoflurane anesthesia.

Animal Experimental Groups

Group 1 - Survival assessment at 3 days.

Animals underwent 30 minutes of ischemic occlusion. Mice treated with CN-105 or vehicle, administered via single intravenous injection 30 minutes after reperfusion (n=12/group). An additional cohort of mice received 2 CN-105 injections (n=12/group), at 30 minutes and 4.5 hours after reperfusion, to determine if repeated dosing could improve survival.

Group 2 - Functional assessment for 7 days

Animals underwent 15 minutes of ischemic occlusion. Mice were treated with CN-105 or vehicle, administered via single intravenous injection at 30 minutes, 2h or 3h after reperfusion. An additional cohort of mice received 2 CN-105 intravenous injections, at 2h and 4h after reperfusion, to determine if repeated dosing could improve functional outcomes. Functional testing included daily rotarod for 7 days and four-limb wire hanging test on day 2 and 5 post-injury (n=12/group and 12 shams).

Group 3 - Infarct volume quantification at 3 days

Two different duration of ischemic occlusion time, namely 30 minutes (n=12/group and 3 shams) and 15 minutes (n=10/group and 3 shams) were used. Both groups were treated with CN-105 or vehicle, administered via single intravenous injection 30 minutes after reperfusion.

Group 4 - Quantitative stereology for F4/80 (microglia) positive cells at 7 days

Animals underwent 15 minutes of ischemic occlusion. Mice treated with either CN-105 or vehicle administered via intravenous injection at 30 min after reperfusion (n= 12/group and 3 shams).

Group 5 - Early differential phosphopeptide expression analysis

All animals underwent 30 minutes of ischemic occlusion. Mice were treated with CN-105 or vehicle administered via intravenous injection 15 minutes after reperfusion. (n= 4/group and 4 shams). Mice were euthanized 15 minutes after injection.

Animal functional assessments

Vestibulomotor performance: Rotarod test

Each mouse was placed on a 3.2 cm diameter rod, with rotation speed increasing from 4 to 40 rpm over 300 seconds. The time in which the mouse was able to stay on the rotating rod before falling (rotarod latency) was determined up to a maximum of 300 seconds. The test was repeated 3 times each day per mouse with an inter-test interval of 15 minutes. The timings were averaged for each day. Training trials were performed daily for 3 consecutive days prior to surgery. Mice not able to achieve at least 200 seconds on the rotarod after 3 days of training were excluded. Baseline rotarod latency was recorded on the day of the surgery. Mice unable to grasp the rotating rod was assigned a latency of 0 seconds.

Motor coordination and limb muscle strength: Four-limb wire hanging test

A setup made of a 35 by 35cm wooden frame fixed with a metal wire grid measuring 0.5 by 0.5cm was used. This frame was mounted 25 cm above a cage with soft bedding to prevent the mice from harming themselves upon falling and discourage them from jumping off the grid intentionally. The mouse was placed in the center of the grid upright prior to the start of the test. The grid was subsequently gently inverted so that the mouse hanged upside down and the timer was started. A maximum of 600 seconds was used. The test was performed at 2 and 7 days post-injury. The test was repeated 3 times with inter-test rest interval of at least 15 minutes. Training trials were performed daily for 3 consecutive days prior to the day of the surgery. Mice unable to attain a hanging latency of 30 seconds on the day of surgery were excluded. Baseline hanging latency was recorded on the day of the surgery. Maximal hanging time was used for analysis. Mice unable to grasp the grid was assigned a latency of 0 seconds.

Contralateral Hemisphere Microglial Quantification

Tissue preparation

An intra-cardiac perfusion of 25 ml of PBS was used. Brains were fixed on 10% formalin for 24 hours at 4°C and transfer to a 30% sucrose-PBS solution for 24 hours at 4°C. Frozen coronal sections of 40 µm were collected on a freezing sliding microtome, distributed in 12 groups and stored at -20C in antifreeze solution (NaHPO4 0.05 M, pH 7.4, 30% Ethylene Glycol, 15% sucrose). The immunostaining was performed using all the section from one group. The tissue

was oxidated with 1% H202 and permeabilized with 0.1% saponin. Activated microglia was detected using anti F4/80 (rat monoclonal, 1:20,000; Serotec, Raleigh, NC) and biotinylated anti-Rat IgG (goat polyclonal, 1:3000; Vector, Burligame, CA). Immuno reaction was developed using ABC kit and peroxidase substrate kit DAB (Vector cat # PK-6100 and SK-4100 Burlingame, CA). The slices were contra-stained with Harleco Hematoxylin (EMD cat # 632-71, Gibbstown, NJ) and cover-slipped using DPX mounting media (Fluka, Milwaukee, WI, USA).

Stereological Analysis

A Nikon 218912 light microscope interfaced with the StereoInvestigator software package (MicroBrightField, Williston, VT, USA) was used. The number of stained cells per mm^3 of hippocampus was estimated by the optical fractionator method, which is an unbiased counting method, independent of the size, shape, and orientation of the cells to be counted. Before counting, all slides were coded to avoid experimenter bias. Before counting, all slides were coded to avoid experimenter bias. As determined by StereoInvestigator, 5 coronal sections (40 μm thick) spaced 12 sections apart were selected along the dorsal hippocampal formation by systematic random sampling. On each section, the whole hippocampal area was delineated using 4x magnification. For microglial quantification, the sampling grid was 500 (X) x 500 (Y) μm, and cells were counted within a probe volume defined by the counting frame (80 x 80 μm) and the dissector height (11 μm). Microglial cells were counted at 20x magnification. Only cells within the counting frame or overlapping the right or superior border of the counting frame, and for which nuclei came into focus while focusing down through the dissector height, were counted.

C8-B4 microglial cells preparation for tumor necrosis factor-alpha measurement.

C8-B4 cells were used at a concentration of 5 x 10^3 cells per well in 96-well plates and incubated overnight with 200μL of Dulbecco's Modified Eagle Medium with High Glucose (DMEM-HG) (Life Technologies, Carlsbad, CA) supplemented with 10% fetal bovine serum non heat-inactivated (FBS non-HI) (HyClone Laboratories, Logan, UT), 100 units/ml penicillin and 100μg/ml streptomycin (Life Technologies, Carlsbad, CA) at 37° C and 5% CO_2. Cells were subsequently incubated overnight in the presence of lipopolysaccharide (LPS) from E. Coli (Sigma-Aldrich, St. Louis, MO) in DMEM-HG and 0.5% FBS non-HI with differing concentrations of CN-105. We investigated the suppression of TNF-α by increasing concentrations of CN-105 (0.1, 0.3 and 1 μM/ml) using fixed LPS stimulation (10ng/ml), and by fixed concentration of 1μM of CN-105 using increasing LPS stimulation (10, 50 and 100ng/ml). All experiments were repeated 6 times and mean TNF-α concentrations were recorded at 4 hours post incubation.

Differential phosphopeptide expression analysis

Sample Preparation

To each hemisphere, 6.6μL/mg 8M urea was added. Samples were subjected one 5-seconds round of tissue tearor and to three 5-seconds rounds of probe sonication with an energy setting of 30%. Samples were then centrifuged at 12,000 g at 4°C for 5 minutes. Protein concentrations were determined by Bradford assay on the supernatant in duplicate (5 μL each assay). Total

protein concentrations ranged from 12.2 mg/mL to 17.8 mg/mL with total protein quantities ranging from 14.7 mg to 21.7 mg. Fourteen mg of each sample was removed and normalized to 14 mg/mL with 50 mM ammonium bicarbonate containing 8M urea. All samples were then reduced for 20 min at 80°C with 10 mM dithiolthreitol and alkylated for 40 minutes at room temperature with 25 mM iodoacetamide. Samples were then diluted 1.6M urea with 50 mM ammonium bicarbonate. Trypsin was added to a 1:25 ratio (enzyme to total protein) and allowed to proceed for 18 hours at 32°C. Samples were then acidified with 0.2% trifluoroacetic acid (TFA) (pH 2.5) and subjected to C18 solid phase extraction (SPE) cleanup (Sep-Pak, 50 mg bed). Following elution, all samples were then frozen and then lyophilized to dryness.

Phosphotyrosine (pY) Antibody Enrichment.

Each entire digested peptide sample was re-suspended in 1.0 mL Immunoaffinity Purification (IAP) Buffer (Cell Signaling Technology) using vortex and brief bath sonication, and transferred to an aliquot of pY- 1000 PTMScan enrichment beads (Cell Signaling Technology). IAP was performed for two hours at 4°C using end-over-end mixing. After spinning to settle the beads and removing the supernatant, pY enrichment beads were washed with three aliquots of IAP buffer and then eluted with two 50 μL aliquots of 0.15% TFA in water, for approximately 10 minutes at room temperature. Eluates were combined and dried via lyophilization. Samples were then spiked with 1000 fmol pre-digested Bovine alpha casein.

Titanium Oxide (TiO2) Enrichment.

Lyophilized peptides were resuspended in 65 μL of 80% acetonitrile/1% TFA. Phosphopeptide enrichments were using p10 GL Bioscience TiO2 spin tips as per a standard protocol (Supplemental data 2). Eluted phosphopeptides were then brought to dryness with lyophilization. Samples were then resuspended in 10 μL of 10 mM citric acid in 0.1%TFA/2% acetonitrile containing 10 fmol/μl yeast alcohol dehydrogenase. To create a "quality control pool" sample to assess analytical reproducibility, 2 μL of each sample was removed and pooled.

Quantitative Analysis of pY phosphoproteome,

Quantitative liquid chromatography–mass spectrometry (LC-MS/MS) was performed on 4 μL of each phosphopeptide enriched sample, using a nanoAcquity UPLC system (Waters Corp) coupled to a Thermo QExactive Plus high resolution accurate mass tandem mass spectrometer (Thermo) via a nanoelectrospray ionization source. Briefly, the sample was first trapped on a Symmetry C18 20 mm × 180 μm trapping column (5 μl/min at 99.9/0.1 v/v water/acetonitrile), after which the analytical separation was performed using a 1.7 μm Acquity BEH130 C18 75 μm × 250 mm column (Waters Corp.) using a 5-minute hold at 3% acetonitrile with 0.1% formic acid and then a 90-min gradient of 3 to 30% acetonitrile with 0.1% formic acid at a flow rate of 400 nanoliters/minute (nL/min) with a column temperature of 55°C. Data collection on the QExactive Plus mass spectrometer was performed in a data-dependent acquisition (DDA) mode of acquisition with a r=70,000 (@ m/z 200) full MS scan from m/z 375 – 1600 with a target AGC value of 1e6 ions followed by 10 MS/MS scans at r-17,500 (@ m/z 200) at a target AGC

value of 5e4 ions. A 20s dynamic exclusion was employed to increase depth of coverage. The total analysis cycle time for each sample injection was approximately 2-hours. Sample order of data collection was interwoven between conditions in order to minimize temporal bias.

Following the 15 LC-MS/MS analyses, data was imported into Rosetta Elucidator v3.3 (Rosetta Biosoftware, Inc), and all LC-MS/MS runs were aligned based on the accurate mass and retention time of detected ions ("features") which contained MS/MS spectra using PeakTeller algorithm (Elucidator) and intensity-scaled based on a robust mean (10%) normalization of the identified pY phosphopeptide features. The relative peptide abundance was calculated based on area-under-the-curve (AUC) of aligned features across all runs. The overall dataset had 78,211 quantified isotope (peptide) groups. Additionally, 612.479 MS/MS spectra were acquired for peptide sequencing by database searching. This MS/MS data was searched against a SwissProt_Mouse database which also contained a reversed-sequence "decoy" database for false positive rate determination as well as Casein_Bovine as a surrogate internal standard. Database searching was performed within Mascot Server (Matrix Science) and annotated at a Mascot ion tolerance of 18.0, which resulted in a peptide false discovery rate of 0.60%. Searching allowed variable M (oxidation, +16 Da), and STY (phosphorylation, +80 Da).

Fold-changes were calculated for CN-105 versus vehicle, as the ratio of the average intensity between the groups. Positive fold-changes mean upregulated in CN-105 versus vehicle, and negative fold-changes mean downregulated in CN-105 versus vehicle and minimum cutoffs were set to 2-fold. Phosphorylated peptides positions were reviewed using the PhosphoSite database[1]. Biological processes and known signaling pathways among the candidate phosphorylated

proteins were classified using Protein Analysis Through Evolutionary Relationships (PANTHERS) version 11.0[2-4] and UniProt database[5].

References

1. Hornbeck PV, Zhang B, Murray B, Kornhauser JM, Latham V, Skrzypek E. PhosphoSitePlus, 2014: mutations, PTMs and recalibrations. Nucleic acids research. 2015 Jan;43(Database issue):D512-20.

2. Mi H, Poudel S, Muruganujan A, Casagrande JT, Thomas PD. PANTHER version 10: expanded protein families and functions, and analysis tools. Nucleic acids research. 2016 Jan 4;44(D1):D336-42.

3. Mi H, Muruganujan A, Casagrande JT, Thomas PD. Large-scale gene function analysis with the PANTHER classification system. Nature protocols. 2013 Aug;8(8):1551-66.

4. Mi H, Thomas PD. Protein Networks and Pathway Analysis. In: Nikolsky Y, Bryant J, editors. Methods in Molecular Biology, Methods and Protocols,2009. p. 123-40.

5. UniProt C. UniProt: a hub for protein information. Nucleic acids research. 2015 Jan;43(Database issue):D204-12.
