# Supplementary material for: Apolipoprotein E mimetic peptide, CN‐105, improves outcomes in ischemic stroke
Source: Ann Clin Transl Neurol. 2017 Mar 9;4(4):246–65. doi: 10.1002/acn3.399 (PMC5376751; doi:10.1002/acn3.399)
Supplement: Supplementary file 3 — Data S3. Supplemental results. [file ACN3-4-246-s003.pdf]

Supplemental material 3: Supplemental Results

A total of 2956 unique phosphopeptides were found in all samples, corresponding to 1152 phosphoproteins.

Enrichment Specificity.

Shown in Supplemental Figure 1 is the percentage of the phosphotyrosine peak area versus total (phosphorylated + non-phosphorylated) peak area. The enrichment varied between 8-37%. This variation can be explained by the low stringency wash steps in the pY and TiOX enrichments. In this experiment, less stringent wash steps produced a high yield of 2956 tyrosine phosphorylated peptides.

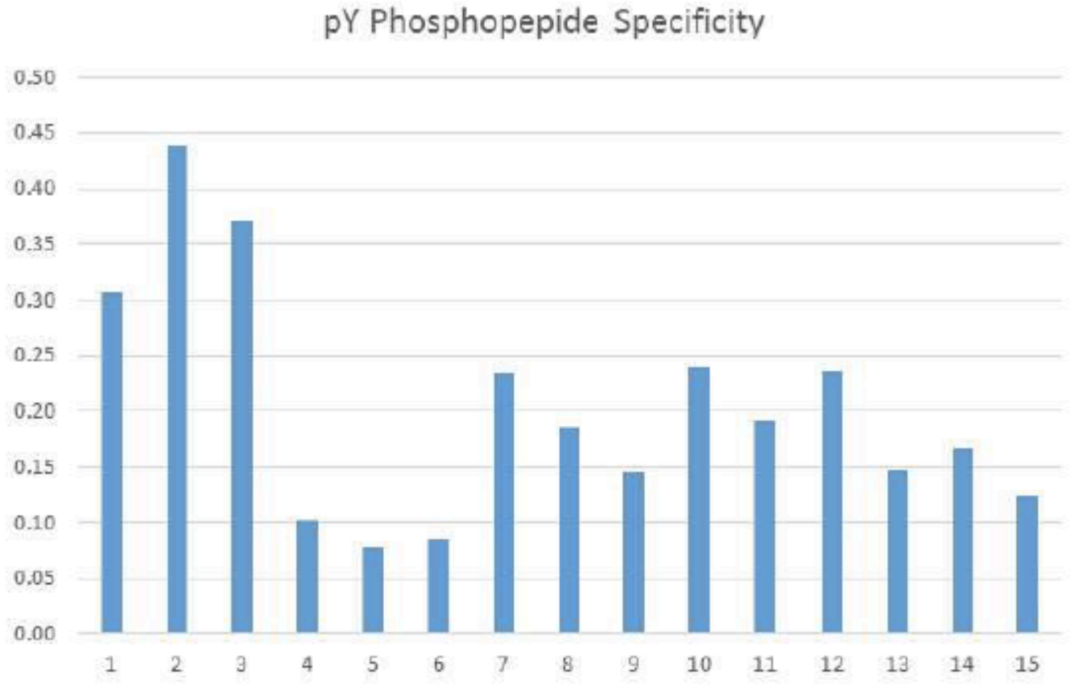

Figure 1. Tyrosine Phosphopeptide specificity (area under the curve) across all samples. 13-15 were QC control samples.

Data Quality Control and Outlier Screening.

To assess system variability, the intensity-scaled phosphopeptide expression data for all identified phosphotyrosine peptides (n=2956) across three quality control (QC) pool samples was calculated to give instrument (LC-MS/MS) system reproducibility. This QC metric resulted in an average relative standard deviation (CV) of 16.9% and a median CV of 10.9% across all 2956 phosphotyrosine peptides. To assessTiO2 enrichment variation, the CV of each of 11 unique phosphopeptides from Casein_Bovine (spiked into the mixture prior to enrichment) was calculated across all 15 analytical injections. The average CV of the casein spiked phosphopeptides was 24%. To measure analytical variation independent of TiO2 enrichment variation, we also spiked each sample with pre-digested yeast ADH post enrichment. The average CV of the 24 measured ADH peptides was 16.4%.

To assess biological variation across the same n=2956 phosphotyrosine peptides, the intensity-scaled phosphopeptide expression data from all identified phosphopeptides within CN105, vehicle, and sham groups were calculated to be 44.7%, 34.6%, and 57.0% respectively. Based on previous phosphoproteomic studies, these levels of biological variation are typical.

To screen for outliers and globally observe the relative contribution of technical and biological variability in the samples, each individual LC-MS/MS analysis was plotted on a PCA plot for the top three principal components (Supplemental Figure 2) based on z-scored transformed log2 phosphopeptide intensity, using all 2956

phosphopeptides measured in the study.

Based on these groupings, there appears to be a global differentiation between the sham group vs the other CN105 or Vehicle groups. The differentiation between vehicle and CN105 was less. As expected, the QC Pool samples were all grouped together towards the center of the distribution.

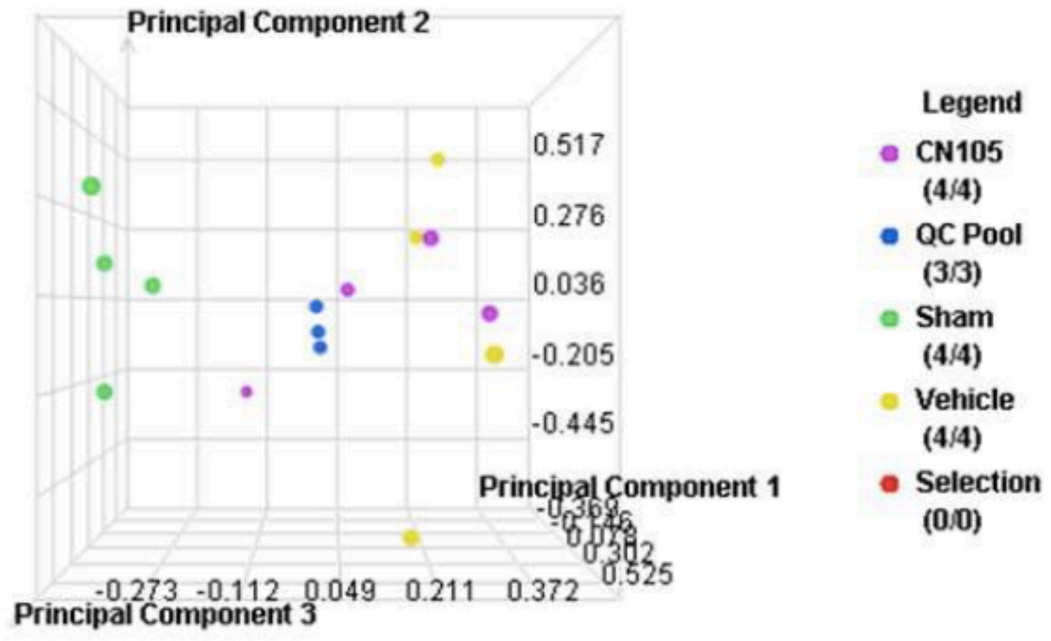

Figure 2. 3D PCA analysis of z-score corrected log2 phosphopeptide intensities including those in QC samples.
