# Supplementary material for: Apolipoprotein E mimetic peptide, CN‐105, improves outcomes in ischemic stroke
Source: Ann Clin Transl Neurol. 2017 Mar 9;4(4):246–65. doi: 10.1002/acn3.399 (PMC5376751; doi:10.1002/acn3.399)
Supplement: Supplementary file 4 — Table S1. Principal biological process of differentially significant phosphoproteins. [file ACN3-4-246-s004.pdf]

Table S1. Principal Biological Process of Differentially Significant Phosphoproteins

Biological Process#	Protein Name	Gene Symbol and UniProtKB number
Cell survival or differentiation processes (15)	Band 4.1-like protein 3	EPB41L3, Q9WV92
	Casein kinase I isoform delta	CSNK1D, Q9DC28
	Dynamin-1-like protein	DNM1L, Q8K1M6
	Eukaryotic translation initiation factor 4 gamma 1	EIF4G1, Q6NZJ6
	Glutathione S-transferase P 1	GSTP1, P19157
	Heat shock protein 105	HSPH1, Q61699
	Myotrophin	MTPN, P62774
	Myotubularin-related protein 5	SBF1, Q6ZPE2
	Peptidyl-prolyl cis-trans isomerase A	PPIA, P17742
	Phosphatidylethanolamine-binding protein 1	PEBP1, P70296
	Programmed cell death 6-interacting protein	PDCD6IP, Q9WU78
	Protein arginine N-methyltransferase 8	PRMT8, Q6PAK3
	Rho GTPase-activating protein 35	ARHGAP35, Q91YM2
	SH3 and PX domain-containing protein 2B	SH3PXD2B, A2AAY5
	Histone H4*	HIST1H4A, P62806
Cell communication and transport (25)	14-3-3 protein beta/alpha	YWHAB, Q9CQV8
	Calcium-dependent secretion activator 2	CADPS2, Q8BYR5
	Cytoplasmic dynein 1 heavy chain 1	DYNC1H1, Q9JHU4
	Dynamin-1	DNM1, P39053
	EH domain-containing protein 3	EHD3, Q9QXY6
	Myosin-Va	MYO5A, Q99104
	Neurochondrin	NCDN, Q9Z0E0
	Probable cationic amino acid transporter	SLC7A14, Q8BXR1
	Protein kinase C and casein kinase substrate in neurons protein 1	PACSIN1, Q61644
	Protein XRP2	RP2, Q9EPK2

	Putative tyrosine-protein phosphatase auxilin	DNAJC6, Q80TZ3
	Receptor-type tyrosine-protein phosphatase-like N	PTPRN, Q60673
	Regulator of G-protein signaling 6	RGS6, Q9Z2H2
	Rho GTPase-activating protein 1	ARHGAP1, Q5FWK3
	SLIT-ROBO Rho GTPase-activating protein 3	SRGAP3, Q812A2
	Src substrate cortactin	CTTN, Q60598
	Synaptic vesicle glycoprotein 2B	SV2B, Q8BG39
	Synaptogyrin-3	SYNGR3, Q8R191
	Syntaxin-binding protein 1	STXBP1, O08599
	Type I inositol 3,4-bisphosphate 4-phosphatase	INPP4A, Q9EPW0
	Vesicular inhibitory amino acid transporter	SLC32A1, O35633
	Disks large-associated protein 2*	DLGAP2, Q8BJ42
	Golgi integral membrane protein 4*	GOLIM4, Q8BXA1
	Probable phospholipid-transporting ATPase IA*	ATP8A1, P70704
	Sodium/potassium-transporting ATPase subunit alpha-1*	ATP1A1, Q8VDN2
Microtubule related functions (5)	Calmodulin-regulated spectrin-associated protein 2	CAMSAP1L1, Q8C1B1
	Calmodulin-regulated spectrin-associated protein 3	KIAA1543, Q80VC9
	CLIP-associating protein 2	CLASP2, Q8BRT1
	Microtubule-associated protein 6	MAP6, Q7TSJ2
	Tubulin polymerization-promoting protein	TPPP, Q7TQD2
Cytoskeletal organization (6)	Ankyrin-2	ANK2, Q8C8R3
	Band 4.1-like protein 1	EPB41L1, Q9Z2H5
	Coronin-1A	CORO1A, O89053
	Coronin-2A	CORO2A, Q8C0P5
	Protein EFR3 homolog B	EFR3B, Q6ZQ18
	Protein FAM126B	FAM126B, Q8C729
Metabolic and homeostasis processes (10)	1-phosphatidylinositol-4,5-bisphosphate phosphodiesterase gamma-1	PLCG1, Q62077
	Alpha-enolase	ENO1, P17182

	Cytochrome c-type heme lyase	HCCS, P53702
	Glutaminase kidney isoform	GLS, D3Z7P3
	Phosphoglycerate kinase 1	PGK1, P09411
	Thioredoxin reductase 1, cytoplasmic	TXNRD1, Q9JMH6
	Triosephosphate isomerase	TPI1, P17751
	UPF0554 protein C2orf43 homolog	LDAH, Q8BVA5
	Wolframin	WFS1, P56695
	Insulin receptor substrate 2*	IRS2, P81122
Immunity (2)	CMRF35-like molecule 8	CD300A, Q6SJQ0
	Tyrosine-protein kinase	LYN, P25911
Unknown (3)	LysM and putative peptidoglycan-binding domain-containing protein 2	LYSMD2, Q9D7V2
	Probable G-protein coupled receptor 158	GPR158, Q8C419
	SH3 and cysteine-rich domain-containing protein 2	STAC2, Q8R1B0

All phosphoproteins listed are down-regulated in CN-105 compared to vehicle in ischemic stroke. The upregulated phosphoproteins are indicated with *.

#Biological function information obtained from Universal Protein Resource (UniProt) at www.uniprot.org on 25th September 2016
