# Supplementary material for: Apolipoprotein E mimetic peptide, CN‐105, improves outcomes in ischemic stroke
Source: Ann Clin Transl Neurol. 2017 Mar 9;4(4):246–65. doi: 10.1002/acn3.399 (PMC5376751; doi:10.1002/acn3.399)
Supplement: Supplementary file 5 — Table S2. Known cell signaling pathways of differentially expressed phosphoproteins. [file ACN3-4-246-s005.pdf]

Table S2. Known Cell Signaling Pathways of Differentially Expressed Phosphoproteins

Protein Name	Gene Symbol and UniProtKB number	Known Pathways Involved^
Insulin receptor substrate 2*	IRS2, P81122	Gonadotropin-releasing hormone receptor pathway<br><br>Insulin/IGF pathway-mitogen activated protein kinase kinase/MAP kinase cascade<br><br>Insulin/IGF pathway-protein kinase B signaling cascade<br><br>Interleukin signaling pathway
1-phosphatidylinositol-4,5-bisphosphate phosphodiesterase gamma-1	PLCG1, Q62077	5HT2 type receptor mediated signaling pathway<br><br>Angiogensis<br><br>Axon guidance mediated by netrin<br><br>CCKR signaling<br><br>EGF receptor signaling<br><br>FGF signaling<br><br>Histamine H1 receptor signaling<br><br>Inflammation mediated by chemokines and cytokines

		Oxytocin receptor mediated signaling<br><br>PDGF signaling<br><br>T cell activation<br><br>Thyrotropin-releasing hormone receptor signaling<br><br>VEGF signaling
Alpha-enolase	ENO1, P17182	Glycolysis
Casein kinase I isoform delta	CSNK1D, Q9DC28	CCKR Signalling<br><br>Circadian Clock System<br><br>Parkinsons disease<br><br>Wnt signaling
Cytoplasmic dynein 1 heavy chain 1	DYNC1H1, Q9JHU4	Huntington Disease
Dynamin-1	DNM1, P39053	Gonadotropin-releasing hormone receptor<br><br>CCKR Signaling
Myosin-Va	MYO5A, Q99104	Nicotinic acetylcholine receptor signaling
Phosphatidylethanolamine-binding protein 1	PEBP1, P70296	FGF signaling

Phosphoglycerate kinase 1	PGK1, P09411	Glycolysis
Protein kinase C and casein kinase substrate in neurons protein 1	PACSIN1, Q61644	Huntington Disease
Regulator of G-protein signaling 6	RGS6, Q9Z2H2	Heterotrimeric G-protein signaling pathway-Gq alpha and Go alpha mediated pathway
Rho GTPase-activating protein 1	ARHGAP1, Q5FWK3	Angiogensis<br><br>Cytoskeletal regulation by Rho GTPase<br><br>PDGF signaling<br><br>VEGF signaling
SLIT-ROBO Rho GTPase-activating protein 3	SRGAP3, Q812A2	PDGF Signaling
Syntaxin-binding protein 1	STXBP1, O08599	Synaptic Vesicle Trafficking
Triosephosphate isomerase	TPI1, P17751	Glycolysis and gluconeogensis
Tyrosine-protein kinase	LYN, P25911	B-cell activation<br><br>Cadherin signaling<br><br>Parkinsons disease

		CCKR signaling

All phosphoproteins listed are down-regulated in CN-105 compared to Vehicle in Ischemic Stroke except those indicated with *.
^Pathways obtained from Protein Analysis Through Evolutionary Relationships (PANTHERS) classification system on www.pantherdb.org on 25th September 2016, version 3.4.1.
