# Supplementary material for: Apolipoprotein E mimetic peptide, CN‐105, improves outcomes in ischemic stroke
Source: Ann Clin Transl Neurol. 2017 Mar 9;4(4):246–65. doi: 10.1002/acn3.399 (PMC5376751; doi:10.1002/acn3.399)
Supplement: Supplementary file 6 — Table S3. Biological functions of phosphoproteins that are significantly different between CN‐105 and vehicle in ischemic stroke. [file ACN3-4-246-s006.pdf]

Table S3. Biological Functions of Phosphoproteins with Statistically Significant Difference Between CN-105 and Vehicle in Ischemic Stroke.

Protein Name	Summary of Protein Biological Function#
1-phosphatidylinositol-4,5-bisphosphate phosphodiesterase gamma-1	Mediates the production of the second messenger molecules diacylglycerol and inositol 1,4,5-trisphosphate.<br><br>Plays an important role in the regulation of intracellular signaling cascades.<br><br>Becomes activated in response to ligand-mediated activation of receptor-type tyrosine kinases.<br><br>Plays a role in actin reorganization and cell migration.
14-3-3 protein beta/alpha	Adapter protein implicated in the regulation of a large spectrum of pathways.<br><br>Blocks the nuclear translocation of the phosphorylated form (by AKT1) of SRPK2 and antagonizes its stimulatory effect on cyclin D1 expression resulting in blockage of neuronal apoptosis elicited by SRPK2.<br><br>Negative regulator of signaling cascades that mediate activation of MAP kinases via AKAP13.
Alpha-enolase	May also function in the intravascular and pericellular fibrinolytic system due to its ability to serve as a receptor and activator of plasminogen on the cell surface of several cell-types such as leukocytes and neurons.<br><br>Stimulates immunoglobulin production
Ankyrin-2	Attaches integral membrane proteins to cytoskeletal elements and also binds to cytoskeletal proteins.
Band 4.1-like protein 1	May function to confer stability and plasticity to neuronal membrane via multiple interactions, including the spectrin-actin-based cytoskeleton, integral membrane channels and membrane-associated guanylate kinases.
Band 4.1-like protein 3	Tumor suppressor that inhibits cell proliferation and promotes apoptosis.<br><br>Modulates the activity of protein arginine N-methyltransferases, including PRMT3 and PRMT5.
Calcium-dependent secretion activator 2	Calcium-binding protein involved in exocytosis of vesicles filled with neurotransmitters and neuropeptides. Probably act upstream of fusion, in the biogenesis or maintenance of mature secretory vesicles.
Calmodulin-	Microtubule minus-end binding protein that may regulate the organization of non-centrosomal microtubules.

regulated spectrin-associated protein 2	May regulate the nucleation and the polymerization of microtubules. Indirectly, through the microtubule cytoskeleton, may regulate the organization of cellular organelles including the Golgi and the early endosomes.
Calmodulin-regulated spectrin-associated protein 3	Microtubule minus-end binding protein that acts as a regulator of non-centrosomal microtubule dynamics and organization.<br><br>May regulate the nucleation and the polymerization of microtubules. Indirectly, through the microtubule cytoskeleton, may regulate the organization of cellular organelles including the Golgi and the early endosomes
Casein kinase I isoform delta	Essential serine/threonine-protein kinase that regulates diverse cellular growth and survival processes including Wnt signaling, DNA repair and circadian rhythms.<br><br>Regulates fast synaptic transmission mediated by glutamate.
CLIP-associating protein 2	Microtubule plus-end tracking protein that promotes the stabilization of dynamic microtubules, is regulated at least in part by phosphatidylinositol 3-kinase signaling.<br><br>Involved in the nucleation of noncentrosomal microtubules originating from the trans-Golgi network (TGN). Required for the polarization of the cytoplasmic microtubule arrays in migrating cells towards the leading edge of the cell.
CMRF35-like molecule 8	Inhibitory receptor which may contribute to the down-regulation of cytolytic activity in natural killer (NK) cells, and to the down-regulation of mast cell degranulation.
Coronin-1A	May be a crucial component of the cytoskeleton of highly motile cells, functioning both in the invagination of large pieces of plasma membrane, as well as in forming protrusions of the plasma membrane involved in cell locomotion.
Coronin-2A	Actin cytoskeleton organization
Cytochrome c-type heme lyase	Links covalently the heme group to the apoprotein of cytochrome c.
Cytoplasmic dynein 1 heavy chain 1	Act as a motor for the intracellular retrograde motility of vesicles and organelles along microtubules.
Disks large-	May play a role in the molecular organization of synapses and neuronal cell signaling. Could be an adapter

associated protein 2	protein linking ion channel to the subsynaptic cytoskeleton.
Dynamin-1	Microtubule-associated force-producing protein involved in producing microtubule bundles and able to bind and hydrolyze GTP. Most probably involved in vesicular trafficking processes and in receptor-mediated endocytosis.
Dynamin-1-like protein	Functions in mitochondrial and peroxisomal division. Through its function in mitochondrial division, ensures the survival of at least some types of postmitotic neurons, including Purkinje cells, by suppressing oxidative damage.<br><br>Required for a normal rate of cytochrome c release and caspase activation during apoptosis. Required for programmed necrosis execution.<br><br>Required for formation of endocytic vesicles.
EH domain-containing protein 3	ATP- and membrane-binding protein that controls membrane reorganization/tubulation upon ATP hydrolysis.<br><br>Plays a role in endocytic transport.
Eukaryotic translation initiation factor 4 gamma 1	Component of the protein complex eIF4F, which is involved in the recognition of the mRNA cap, ATP-dependent unwinding of 5'-terminal secondary structure and recruitment of mRNA to the ribosome.
Glutaminase kidney isoform	Regulates the levels of the neurotransmitter glutamate in the brain
Glutathione S-transferase P 1	Conjugation of reduced glutathione to a wide number of exogenous and endogenous hydrophobic electrophiles.
Golgi integral membrane protein 4	Plays a role in endosome to Golgi protein trafficking.
Heat shock protein 105	Prevents the aggregation of denatured proteins in cells under severe stress, on which the ATP levels decrease markedly. Inhibits HSPA8/HSC70 ATPase and chaperone activities.
Histone H4	Histones play a central role in transcription regulation, DNA repair, DNA replication and chromosomal

	stability.
Insulin receptor substrate 2	May mediate the control of various cellular processes by insulin.
LysM and putative peptidoglycan-binding domain-containing protein 2	Unknown
Microtubule-associated protein 6	Involved in microtubule stabilization in many cell types, including neuronal cells.
Myosin-Va	Processive actin-based motor that can move in large steps approximating the 36-nm pseudo-repeat of the actin filament.<br><br>Involved in melanosome transport. Also mediates the transport of vesicles to the plasma membrane.
Myotrophin	Promotes dimerization of NF-kappa-B subunits and regulates NF-kappa-B transcription factor activity.<br><br>Plays a role in the regulation of the growth of actin filaments.
Myotubularin-related protein 5	Probable pseudophosphatase.<br><br>May function as a guanine nucleotide exchange factor (GEF).
Neurochondrin	Probably involved in signal transduction, in the nervous system.
Peptidyl-prolyl cis-trans isomerase A	PPIases accelerate the folding of proteins.
Phosphatidylethanolamine-binding protein 1	Binds ATP, opioids and phosphatidylethanolamine.<br><br>Serine protease inhibitor which inhibits thrombin, neuropsin and chymotrypsin but not trypsin, tissue type plasminogen activator and elastase.<br><br>Inhibits the kinase activity of RAF1 by inhibiting its activation and by dissociating the RAF1/MEK complex and acting as a competitive inhibitor of MEK phosphorylation.

	May be involved in the function of the presynaptic cholinergic neurons of the central nervous system.
Phosphoglycerate kinase 1	In addition to its role as a glycolytic enzyme, it seems that PGK-1 acts as a polymerase alpha cofactor protein (primer recognition protein).
Probable cationic amino acid transporter	May be involved in arginine transport.
Probable G-protein coupled receptor 158	Orphan receptor.
Probable phospholipid-transporting ATPase IA	Catalytic component of a P4-ATPase flippase complex; Transport of aminophospholipids from the outer to the inner leaflet of various membranes and ensures the maintenance of asymmetric distribution of phospholipids. Acts as aminophospholipid translocase at the plasma membrane in neuronal cells; the activity is associated with hippocampus-dependent learning.
Programmed cell death 6-interacting protein	Involved in concentration and sorting of cargo proteins of the multivesicular body (MVB) for incorporation into intralumenal vesicles that are generated by invagination and scission from the limiting membrane of the endosome.<br><br>Required for completion of cytokinesis.<br><br>May play a role in the regulation of both apoptosis and cell proliferation.
Protein arginine N-methyltransferase 8	Membrane-associated arginine methyltransferase
Protein EFR3 homolog B	Component of a complex required to localize phosphatidylinositol 4-kinase (PI4K) to the plasma membrane.
Protein FAM126B	Component of a complex required to localize phosphatidylinositol 4-kinase (PI4K) to the plasma membrane.
Protein kinase C and casein kinase substrate in neurons	Binds to membranes and mediates membrane tubulation. Plays a role in the reorganization of the microtubule cytoskeleton.

protein 1	Plays a role in cellular transport processes by recruiting DNM1, DNM2 and DNM3 to membranes.<br><br>Plays a role in the regulation of neurite formation, neurite branching and the regulation of neurite length.<br><br>Required for normal synaptic vesicle endocytosis; this process retrieves previously released neurotransmitters to accommodate multiple cycles of neurotransmission.<br><br>Required for normal excitatory and inhibitory synaptic transmission.
Protein XRP2	Acts as a GTPase-activating protein (GAP) involved in trafficking between the Golgi and the ciliary membrane.<br><br>Involved in localization of proteins to the cilium membrane.
Putative tyrosine-protein phosphatase auxilin	Plays a role in clathrin-mediated endocytosis in neurons; recruits HSPA8/HSC70 to clathrin-coated vesicles and promotes uncoating of clathrin-coated vesicles.
Receptor-type tyrosine-protein phosphatase-like N	Plays a role in vesicle-mediated secretory processes.<br><br>Required for normal accumulation of secretory vesicles in hippocampus.<br><br>Required for normal accumulation of the neurotransmitters norepinephrine, dopamine and serotonin in the brain<br><br>Seems to lack intrinsic enzyme activity
Regulator of G-protein signaling 6	Regulates G protein-coupled receptor signaling cascades.
Rho GTPase-activating protein 1	GTPase activator for the Rho, Rac and Cdc42 proteins, converting them to the putatively inactive GDP-bound state.
Rho GTPase-activating protein 35	Represses transcription of the glucocorticoid receptor. May transduce signals from p21-ras to the nucleus, acting via the ras GTPase-activating protein (GAP)

SH3 and cysteine-rich domain-containing protein 2	Unknown function
SH3 and PX domain-containing protein 2B	Adapter protein involved in invadopodia and podosome formation and extracellular matrix degradation.
SLIT-ROBO Rho GTPase-activating protein 3	GTPase-activating protein for RAC1 and perhaps CDC42; may attenuate RAC1 signaling in neurons.
Sodium/potassium-transporting ATPase subunit alpha-1	This is the catalytic component of the active enzyme, which catalyzes the hydrolysis of ATP coupled with the exchange of sodium and potassium ions across the plasma membrane.
Src substrate cortactin	Contributes to the organization of the actin cytoskeleton and cell shape, and cell migration.<br><br>Plays a role in the regulation of neuron morphology, axon growth and formation of neuronal growth cones<br><br>Plays a role in intracellular protein transport and endocytosis, and in modulating the levels of potassium channels present at the cell membrane.<br><br>Plays a role in receptor-mediated endocytosis via clathrin-coated pits.
Synaptic vesicle glycoprotein 2B	Probably plays a role in the control of regulated secretion in neural and endocrine cells. Receptor for the botulinium neurotoxin type A/BOTA
Synaptogyrin-3	Involved in the positive regulation of dopamine transporter activity.
Syntaxin-binding protein 1	May participate in the regulation of synaptic vesicle docking and fusion, possibly through interaction with GTP-binding proteins. Essential for neurotransmission and binds syntaxin.
Thioredoxin reductase 1, cytoplasmic	Plays a role in selenium metabolism and protection against oxidative stress.
Triosephosphate	Glycolysis and gluconeogensis

isomerase	
Tubulin polymerization-promoting protein	May play a role in the polymerization of tubulin into microtubules, microtubule bundling and the stabilization of existing microtubules.<br><br>May play a role in mitotic spindle assembly and nuclear envelope breakdown.
Type I inositol 3,4-bisphosphate 4-phosphatase	Involved in the regulation of megakaryocyte, fibroblast proliferation and regulates cell growth.
Tyrosine-protein kinase	Non-receptor tyrosine-protein kinase that transmits signals from cell surface receptors and plays an important role in the regulation of innate and adaptive immune responses, hematopoiesis, responses to growth factors and cytokines, integrin signaling, but also responses to DNA damage and genotoxic agents.<br><br>Required for the initiation of the B-cell response, but also for its down-regulation and termination.<br><br>Plays a role in the inflammatory response to bacterial lipopolysaccharide.<br><br>Mediates the responses to cytokines and growth factors in hematopoietic progenitors, platelets, erythrocytes, and in mature myeloid cells, such as dendritic cells, neutrophils and eosinophils.<br><br>Plays an important role in integrin signaling. Regulates cell proliferation, survival, differentiation, migration, adhesion, degranulation, and cytokine release.<br><br>Down-regulates signaling pathways by phosphorylation of immunoreceptor tyrosine-based inhibitory motifs (ITIM).<br><br>Regulates activation of the MAP kinase signaling cascade, including activation of MAP2K1/MEK1, MAPK1/ERK2, MAPK3/ERK1, MAPK8/JNK1 and MAPK9/JNK2.
UPF0554 protein C2orf43 homolog	Serine lipid hydrolase associated with lipid droplets, could promote cholesterol ester turnover in macrophages.

Vesicular inhibitory amino acid transporter	Involved in the uptake of GABA and glycine into the synaptic vesicles.
Wolframin	Participates in the regulation of cellular Ca2+ homeostasis, at least partly, by modulating the filling state of the endoplasmic reticulum Ca2+ store

* Underlined amino acid represents phosphorylation for tyrosine and cysteine, and oxidation for methionine.
#Biological function information obtained from Universal Protein Resource (UniProt) at www.uniprot.org on 25th September 2016
^Pathway information obtained from Protein Analysis Through Evolutionary Relationships (PANTHERS) classification system on www.pantherdb.org on 25th September 2016, version 3.4.1.
